# Mechanical characterization of elastic edible films with single, crosslinked and interpenetrating biopolymer networks using combined uniaxial and biaxial analysis

**DOI:** 10.1016/j.crfs.2026.101381

**Published:** 2026-03-13

**Authors:** Quinten Steffens, Dyllan Gan Yu-Jing, Naomi Schuppert, Ruud van der Sman, Remko Boom, Yizhou Ma, Lu Zhang

**Affiliations:** a Laboratory of Food Process Engineering, Wageningen University & Research, Bornse Weilanden 9, WG Wageningen, 6708, the Netherlands; b Food & Biobased Research, Wageningen University & Research, Bornse Weilanden 9, WG Wageningen, 6708, the Netherlands; c Laboratory of Food Quality and Design, Wageningen University & Research, Bornse Weilanden 9, WG Wageningen, 6708, the Netherlands; d Food Science Department, University of Copenhagen, Rolighedsvej 26, Frederiksberg, 1958, Denmark

**Keywords:** Edible films, Tensile strength, Plasticizer, Computer vision

## Abstract

Stretchable and compliant films can be prepared from edible biopolymers such as hydrocolloids and proteins. Their mechanical performance under stress affects their potential applications. This study investigates the uniaxial and biaxial extensional properties of various edible hydrogel films plasticized to various degrees. Hydrogel films consisting of single biopolymer networks (SN), crosslinked networks (CN) and interpenetrating networks (IPN) were prepared using gelatine, gelatine-caseinate and alginate-agar, respectively, with different concentrations of glycerol. Their extensional properties were characterized with computer-vision-based biaxial extension testing and compared to standard uniaxial tensile testing. The IPN films exhibited strain-hardening and the highest stiffness and tensile strength. Adding plasticizer reduced the stiffness of all film types, and also reduced the tensile strength in SN and CN films. The influence of the plasticizer on the tensile strength of IPNs is governed by the architectures of the first and second networks. While standard, uniaxial extension of IPN films showed no relation between glycerol content and fracture properties, our biaxial methods showed that plasticization by glycerol reduced both fracture strain and stress. Our results highlighted the necessity of combining uniaxial and biaxial tensile tests to evaluate film properties for practical applications relying on different types of stress load. The insight obtained may be used to design edible films with improved extensional performance, and our biaxial method could potentially be used as a screening tool for evaluating tensile properties in novel materials.

## Highlights

• Plasticizers decrease elastic modulus and tensile strength of hydrogel films.
• Interpenetrating network films respond differently to uniaxial and biaxial loads.
• Interpenetrating network films possess very high tensile strength.
• Bulge deformation is more accurately approximated by ellipsoids than spheres.
• Combined uniaxial and biaxial tensile tests offer a more complete insight in film behaviour.

## Graphical abstract

**Figure:**
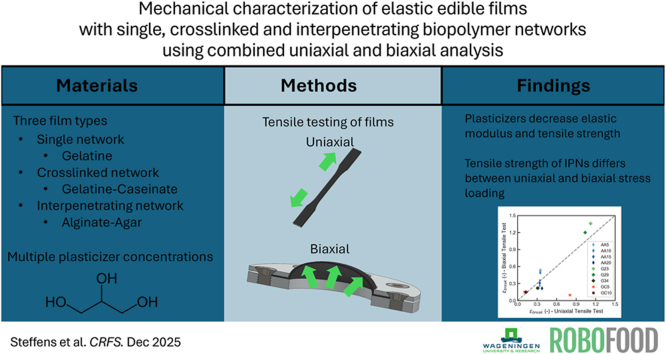

**Table:** Nomenclature

*a, b*	Half-major and half-minor axis length of ellipse (m)
***A, A*_*0*_**	Instantaneous and initial cross-sectional area (m^2^)
***F***	Force (n)
***h, h*_*0*_**	Instantaneous and initial gauge length (m)
***h*_*b*_**	Bulge height from the film clamp to the apex of the bulge (m)
***K***	Strength coefficient (Pa)
***n***	Strain hardening exponent (−)
***p***	Pressure (Pa)
***R*_*1*_**, ***R*_*2*_**	Radius of curvature (m)
***S, S*_*0*_**	Instantaneous and initial film thickness (m)
$\varepsilon_{1},\varepsilon_{2}$	True strains in principal directions 1 and 2 (−)
$\varepsilon_{3}$	True thickness strain (−)
$\sigma_{1},\sigma_{2}$	True principal stresses in principal directions 1 and 2 (Pa)
$\sigma_{B}$	Equi-biaxial true stress (Pa)

## 1 Introduction

Growing concerns from consumers and governments about sustainability and safety of plastics films based on petroleum-derived polymers have caused increasing interest in the development of polymer films based on edible materials (Khalesi et al., 2020; Leceta et al., 2014; Yin et al., 2024). Although edible films have excellent biodegradability and biocompatibility, their mechanical performance requires further improvement (Hartmann et al., 2021; Nath et al., 2022; Walker et al., 2017; Wittaya, 2012). Adoption of edible films in food and pharmaceutical packaging, tissue engineering, and soft robotics, requires a better assessment of their elasticity and tensile strength.

Mechanical properties of edible films, such as elasticity and tensile strength, are affected by the type of polymer network and can be modulated by plasticization. Edible single network (SN) films contain a single film-forming biopolymer, such as a protein or a hydrocolloid. Single-network biopolymers generally exhibit limited stretchability, with gelatine being a notable exception due to reversible crosslinking by triple helices that can dissipate stresses during stretching (Baumgartner et al., 2020; Edward and Golecki, 2023; Shintake et al., 2017). Crosslinked network (CN) films contain at least two film-forming polymers with a single, intermolecularly (often covalently) crosslinked network that improves the film elasticity and robustness (Guo et al., 2014; Khalesi et al., 2020). Interpenetrating polymer network (IPN) films also contain at least two polymers, but these are sequentially crosslinked to form two distinct polymer networks that are molecularly intertwined but not covalently bonded, resulting in enhanced strength and flexibility by combining a rigid network with a soft, sacrificial one (Bootsma et al., 2017; Jeong et al., 2020; Lin et al., 2019; Suo et al., 2018). The polymer networks can be formed by hydrocolloids, which contain many hydroxyl groups and often are polyelectrolytes as well (e.g. alginate), fibrous proteins such as gelatine, and globular proteins such as casein (Baumgartner et al., 2020; Edward and Golecki, 2023; Janes and Dai, 2012; Khwaldia et al., 2004).

The mechanical properties of films can be further modified through the incorporation of plasticizers. Plasticizers enhance the chain mobility, which increases the maximum strain at break but often also reduces the tensile strength and Young's modulus (Gao et al., 2017; Lawton, 2004; Yoo and Krochta, 2011). Glycerol is particularly effective but may compromise the gel stability (L. Li et al., 2018). Plasticizers may also improve the compatibility between multiple polymers, enhancing the physical stability of DN and IPN films (Brindle and Krochta, 2008; L. Li et al., 2018). Thus, there are many variables affecting the mechanical properties of edible films and optimizing mechanical properties for specific applications is therefore not trivial. Quantitative evaluation of mechanical properties may help to accelerate the formulation development.

To evaluate the effect of the network structure and plasticizer content on the tensile properties of edible thin films, characterization of the extensional properties can be performed by applying a load to a sample and measuring its stress-strain response in tensile tests. Small deformation properties such as Young's modulus and large deformation properties such as tensile strength and elongation at break can be obtained from standardized uniaxial tensile tests (Kozempel and Tomasula, 2004; Yoo and Krochta, 2011). However, in practical applications polymer films generally undergo biaxial deformation. For example, packaging films typically are wrapped/spread over and around a product and in that process are extended biaxially. Despite this, a comprehensive comparison between the influence of plasticizer content and network type on the uniaxial versus biaxial tensile properties of edible films has not yet been reported.

Biaxial deformation can be characterized using a bulge test, which involves fixating a circular film over a cylindrical pressure chamber and deforming the film by applying a pressure difference over the film. This pressure differential causes biaxial deformation, which in turn can be quantified using an optical measurement system, and related to the momentary pressure difference. Thus a force-deformation curve can be obtained, which can be converted to a stress-strain curve and compared to uniaxial testing. Since the stress distribution over a thin film is fundamentally different for uniaxial and biaxial deformation, the force-deformation curves are different as well, and since applications typically involve biaxial deformation, it is important to investigate this difference. Biaxial loading also allows the use of larger strain ranges before material failure (Ebrahimi and Lotfabad, 2022; Hallfeldt et al., 2014). Combining uniaxial with biaxial tensile tests is thus beneficial as this practice provides a more complete characterization of film properties.

Therefore, this study investigates the tensile properties of edible films using uniaxial and biaxial tensile tests, on a range of different films, by varying the type of polymeric network and the degree of plasticization. Edible films based on gelatin, gelatin-caseinate, and alginate-agar represent three network types, SN, CN, and IPN, and were each formulated with a range of plasticizer levels. Standardized uniaxial tensile test were performed to determine the small and large extensional properties using a universal testing machine. A standardized biaxial tensile tester known as the alveograph is used in the food industry to measure empirical dough properties as indicators of flour quality (Dufour et al., 2024; Jødal and Larsen, 2021). However, alveographs do not provide full stress-strain curves. Therefore, a bulge-test based method was developed to characterize the biaxial tensile properties of the films. This computer-vision based method includes a novel image processing and analysis workflow to convert pressure-deformation data to full stress-strain curves. Finally, the large deformation properties obtained using uniaxial and biaxial tests were compared to provide comprehensive insight on the tensile properties of edible films. The approach described in this study may serve as the basis for future investigations aiming to inform the design of edible polymer films with mechanical properties optimised for packaging, tissue engineering and soft robotics.

## 2 Materials and methods

### 2.1 Materials

Agar, calcium chloride dihydrate (purity >99%), citric acid (purity ≥97%) and sodium alginate were purchased from Sigma-Aldrich (Zwijndrecht, the Netherlands). Gelatine (bloom value 116) and glycerol (purity 99.6%) were obtained from VWR Chemicals (The Netherlands). Microbial transglutaminase (Activa WM, nominal activity 100U/g) was purchased from Ajinomoto (Nesle, France). Sodium caseinate (dry matter content of 48.5% w/w) was provided by FrieslandCampina (Wageningen, The Netherlands). Food colouring (Wilton) and glucose syrup (Brand New Cake) were purchased from online retailers.

### 2.2 Edible film preparation

Edible films with three types of network structures were prepared by preparing film forming solutions, drying and conditioning.

Solutions to prepare single-network (SN) gelatine films were formulated as reported by Baumgartner et al. (2020). Three samples were prepared using 23, 29 and 34% (w/w) glycerol as plasticizer and were abbreviated as G23, G29, and G34, respectively (Table 1). First, citric acid (3.6% w/w), glucose syrup (25% w/w), and glycerol were dissolved in deionized water at 70 °C under continuous stirring at 400 RPM for at least 1 h. Afterwards, the solution was cooled to 25 °C. Second, gelatine was added while the solutions were stirred at 50 RPM for at least 1 h. The solution was then heated to 70 °C at 400 RPM and immediately deaerated in a vacuum mixer (ARV-310P, Thinky, Japan) at 2350 rpm, 350 mbar for 2 min.

**Table 1:** Formulations of edible films: single network (SN), crosslinked network (CN), and interpenetrating network (IPN).

Component (%/w/w) a	SN: Gelatine			CN: Gelatine-Caseinate			IPN: Alginate-Agar b			
	*G23*	*G29*	*G34*	*GC5*	*GC10*	*GC15*	*AA5*	*AA10*	*AA15*	*AA20*
Deionized water	34	28	23	72	77	82	76	81	86	91
Glycerol	23	29	34	5	10	15	5	10	15	20
Glucose syrup	25	25	25	-	-	-	-	-	-	-
Gelatine	14	14	14	1.6	1.6	1.6	-	-	-	-
Citric acid	3.6	3.6	3.6	-	-	-	-	-	-	-
Caseinate	-	-	-	10	10	10	-	-	-	-
Microbial transglutaminase	-	-	-	0.9	0.9	0.9	-	-	-	-
Alginate	-	-	-	-	-	-	0.9	0.9	0.9	0.9
Agar	-	-	-	-	-	-	3.0	3.0	3.0	3.0

a weight percentage of each component in the film forming solutions (i.e. before drying/casting of the film).

b CaCl_2_ solution was used to crosslink alginate.

Solutions to prepare crosslinked-network (CN) edible films were formulated using conjugates of gelatine and sodium caseinate as described by Chambi and Grosso (2006). Three sample were prepared using 5, 10, and 15% (w/w) glycerol as plasticizer, which were abbreviated as GC5, GC10, and GC15, respectively (Table 1). Gelatine (1.6% w/w) was hydrated in deionized water at room temperature for at least 1 h. Subsequently, sodium caseinate (10% w/w) and glycerol were added while the mixture was stirred at 400 RPM and 60 °C for an hour. Microbial transglutaminase (0.88% w/w) was added to crosslink for 15 min and deactivated by heating to 85 °C. A sample solution of GC10 without transglutaminase was prepared as a control to verify whether the crosslinking was successful in other samples.

The solutions to prepare SN and CN edible films were stained with a black dye, subsequently poured into petri dishes coated with sunflower oil as release agent and dried in a fume hood for 24 h.

Solutions to prepare interpenetrating-network (IPN) films were formulated using composites of sodium alginate and agar as described by Li et al. (2018). Four levels of glycerol (5, 10, 15, and 20% w/w) were used as plasticizer. Four sample were prepared using 5, 10, 15 and 20% (w/w) glycerol as plasticizer, which were abbreviated as AA5, AA10, AA15 and AA20, respectively (Table 1). Sodium alginate (0.9% w/w), agar (3% w/w), a black dye and glycerol were dissolved in deionized water at 95 °C under continuous stirring at 400 RPM for at least 1 h (Table 1). The solutions were poured directly into a Petri dish coated with sunflower oil and dried for 24 h inside a fume hood. Next, 1M CaCl_2_ solution was poured onto the dried films to crosslink the sodium alginate for 4 h. After the treatment, the films were taken out of the petri dishes and any excessive liquid were removed with paper towel. A sample solution of AA10 for which CaCl_2_ steeping was omitted was prepared as a control to verify whether IPN formation was successful in other samples.

Prior to mechanical testing, all films were conditioned at 20 °C and 50% relative humidity until the mass loss between subsequent measurements at 24 h intervals was less than 1%.

### 2.3 Uniaxial tensile testing

**Fig. 1:**
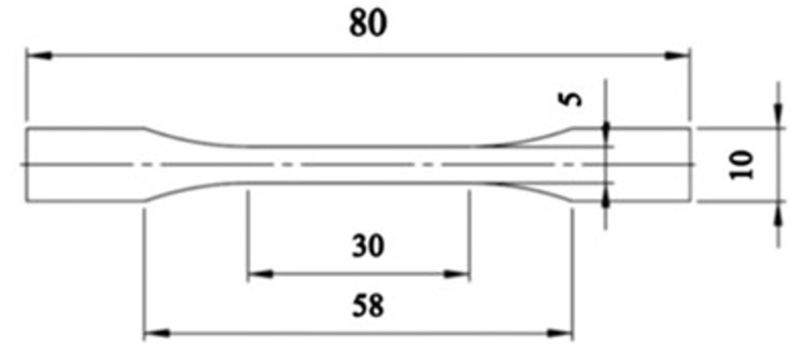
Dimensions (in mm) of samples used for uniaxial tensile testing.

Dog bone shaped samples (n = 5 per sample) as defined by ISO 527-2 shape 1BA were cut from conditioned films using a hydraulic press (Fig. 1). The sample thickness and width were measured at the end and middle of the rectangular section of the samples using a digital calliper to calculate the cross-sectional area of the sample (Mitutoyo Manufacturing Co. Tld., Japan). Uniaxial tensile tests were performed using a Zwick Z010 tensile tester according to ISO 527-2 protocol. The tester was equipped with pneumatic grips (type 8195) with integrated control valves to maintain constant pressure during testing. A load cell of 100N was used and the initial clamp distance was set to 55 mm. The strain was measured using a clip-on extensometer that contacted the sample at two points 25 mm apart. The testing speed was 100 mm/min, with the exception of the IPN samples where the testing speed was reduced to 10 mm/min due to the high tensile strength of the samples.

The force and displacement were recorded with the testXpert software (Zwick Roell, Venlo, The Netherlands). True stress $\sigma_{True}$ (Pa) was calculated under the assumption of incompressibility as

(1) $$\sigma_{True}=\frac{F}{A}$$

With *F* the force (n) and *A* the area (m^2^). The cross-sectional area is dependent on the instantaneous elongation and is given by

(2) $$A=\frac{h_{0}}{h}A_{0}$$

With *h*_*0*_ the initial gauge length (mm), *h* the current gauge length and *A*_*0*_ the initial cross-sectional area of the sample (mm^2^). The true strain $\varepsilon_{True}$ (−) was calculated as

(3) $$\varepsilon_{True}=\ln\left(\frac{h}{h_{0}}\right)$$

Young's modulus was calculated as the slope of the stress-strain curve in the linear elastic region between 0.05 and 0.5% of strain (for statistical details, refer to ‘Young's modulus' section of the Supplementary Materials). Strain and stress at break were determined at the peak stress of the true strain-stress curve.

The true strain and stress during plastic deformation were fitted to the Hollomon Law:

(4) $$\sigma_{True}=K\varepsilon_{True}^{n}$$

With *K* the strength coefficient (Pa) and *n* (−) the strain hardening exponent (Ebrahimi and Lotfabad, 2022; Li et al., 2019).

### 2.4 Biaxial bulge testing

#### 2.4.1 Bulge test principles and deriving true stress-strain curves

Biaxial deformation is a different phenomenon relative to uniaxial deformation, and thus it is useful to investigate this, next to uniaxial deformation. Bulge testing is however also advantageous over uniaxial testing as it allows the characterization of materials exhibiting significant plastic deformation, since higher strain levels can be achieved before fracture occurs and the method is less sensitive to necking (Dudderar et al., 1977; Ebrahimi and Lotfabad, 2022; Güner et al., 2009; Kaya et al., 2008). However, reports of bulge tests on (edible) soft films are scarce, despite their relevance to applications in for example packaging, tissue engineering, and soft robotics (Huang et al., 2007).

**Fig. 2:**
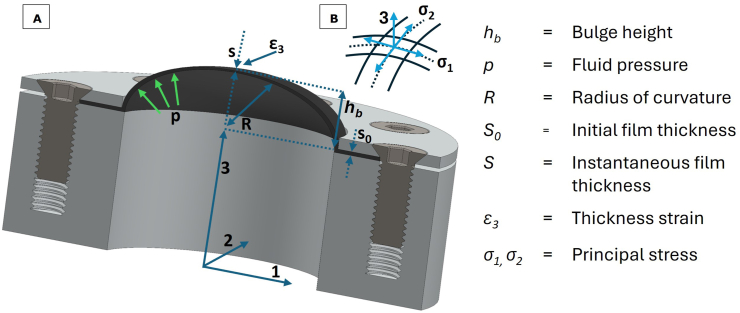
A) Schematic representation of a section of a deformed sample clamped in the bulge test setup B) Direction of principal stresses $\sigma_{1}$ and $\sigma_{2}$ at the apex of the bulged film.

In bulge testing a flat film of a sample is fixated onto a die having a circular orifice, and clamped around the perimeter using a sample holder (Fig. 2). An elevated pressure *p* is gradually applied to one side of the sample, causing it to bulge to the other side. As the test conditions are nearly frictionless, the true stress at the apex of the bulge can subsequently be calculated from the applied pressure, the local radius of curvature and the thickness of the sample (raw data are provided in the ‘Film thickness’ section of the Supplementary Materials).

The local radius of curvature can be derived from images captured with a sideways camera as function of time (Hallfeldt et al., 2014; Mulder et al., 2015; Stachowicz and Rzeszowska, 2003). However, in-situ measurement of the local thickness of films at their apex during bulge testing is not trivial. Different relations for deriving the local thickness at the film apex as function of local deformation have been proposed (Min et al., 2017). These are based on geometric constraints or on empirical observation, e.g. by freezing bulged samples (Charalambides et al., 2002; Ebrahimi and Lotfabad, 2022; Kruglov et al., 2002; Lazarescu et al., 2011; Stachowicz and Rzeszowska, 2003).

To derive the local thickness of the apex it is often assumed that the deformation is uniform over the bulge, in which case the bulge shape can be approximated by a sphere (Charalambides et al., 2002; Hallfeldt et al., 2014; Hill, 1950). However, at the perimeter, the film is constrained by a clamp, and as such can only deform uniaxially in the direction orthogonal to the perimeter. At the top apex of the bulge, the film is stressed purely biaxially and is thus stretched thinner than at the rim. An extra degree of freedom is required during fitting of the bulge shape to accommodate for these constraints. During bulge testing of metals a superior fit was provided with a spheroid (an axisymmetric ellipse) compared to a sphere (an axisymmetric circle), which provides the extra degree of freedom (Ebrahimi and Lotfabad, 2022; Kaya et al., 2008; Mulder et al., 2015). Due to the axial radial symmetry, the principal curvatures $R_{1}$ and $R_{2}$ (Fig. 2) at the apex are equal and the film deforms equibiaxially, thus the stress in both principal directions around the apex must be equal: $\sigma_{1}=\sigma_{2}=\sigma_{b}$ (Hallfeldt et al., 2014; Mulder et al., 2015). Min et al. showed that even if $R_{2}$/$R_{1}$ is larger than ∼0.5 the stress ratio $\sigma_{1}/\sigma_{2}$ is still larger than 0.99, as long as the radius of principal curvature is > 50 times larger than the film thickness (2017). For this equibiaxial stress state at the apex, the force equilibrium equation for thin shells equals (Mulder et al., 2015; Stachowicz and Rzeszowska, 2003):

(5) $$\sigma_{b}=\frac{\mathrm{pR}}{2s}$$

With $\sigma_{b}$ the biaxial true stress (Pa), p (Pa) is the pressure, *R* the curvature radius (m), and *s* the thickness of the film (m). The radius of curvature at the bulge apex is given by:

(6) $$R_{1}=\frac{b^{2}}{a}$$

**Fig. 3:**
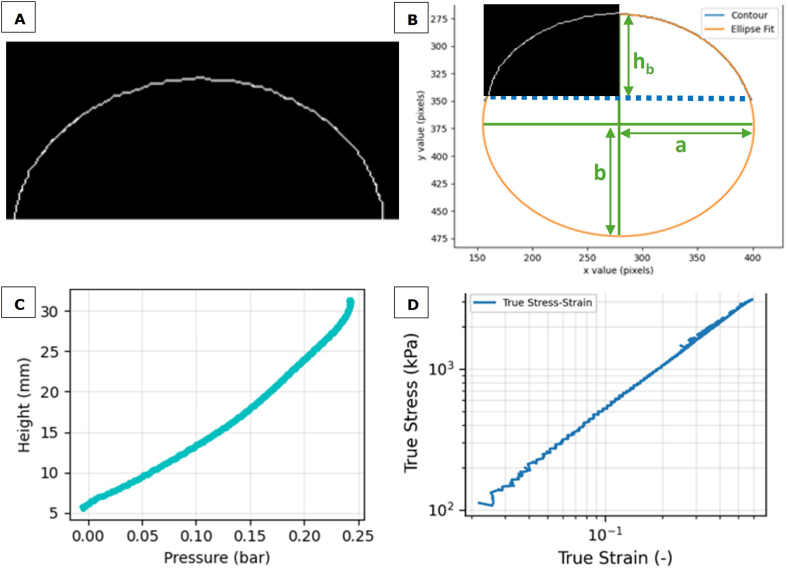
A) Bulge outline frame after application of filtering steps; B) Ellipse fit generated during image processing, with the left half of the frame from A overlaid as visual guide. The bulge outline is represented by a blue line, the fitted ellipse by an orange line and the film clamp by a dashed blue line. The green arrows indicate the major axis 2a, the minor axis 2b and the height h_b_ from the film clamp to the apex of the bulge; C) Pressure-height curve of a representative sample; D) True stress-strain curve of the same representative sample.

With b the semi-minor axis length (m) and a the semi-major axis length (m) as determined from the ellipse fit during the bulge test (Fig. 3B).

Because of volume preservation the true through-thickness strain $\varepsilon_{3}$ equals (Hallfeldt et al., 2014):

(7) $$\varepsilon_{3}=\ln\left(\frac{s}{s_{0}}\right)=-\left(\varepsilon_{1}+\varepsilon_{2}\right)$$

With $\varepsilon_{3}$ the true strain (−) and $s_{0}$ the thickness of the film in unstressed state. Thus we can calculate the instantaneous film thickness as (Charalambides et al., 2002; Min et al., 2017):

(8) $$s=s_{0}\;e^{-\left(\varepsilon_{1}+\varepsilon_{2}\right)}=s_{0}\;e^{-2\varepsilon_{b}}$$

By combining equations (5) and (8), a relation between the biaxial strain and stress is obtained:

(9) $$\sigma_{b}=\frac{\mathrm{pR}}{2s_{0}}e^{2\varepsilon_{b}}$$

Mulder et al. (2013) proposed that the strain hardening exponent from the Hollomon law.

(equation 4, $\sigma_{True}=K\cdot \varepsilon_{True}^{n}$), obtained by uniaxial testing, may be used to calculate $\varepsilon_{b}$. We borrowed inspiration from this idea. The von-Mises equivalent true strain $\bar{\varepsilon }$ can be used to predict the stress under biaxial loading conditions from uniaxial tensile test data. With $\varepsilon_{1}=\varepsilon_{2}=\varepsilon$ and $\varepsilon_{3}=-2\varepsilon$ the von-Mises strain equivalent true strain $\bar{\varepsilon }$ becomes (Rees, 1995) becomes:

(10) $$\bar{\varepsilon }=\sqrt{\frac{2}{3}\left[{\left(\varepsilon_{1}-\varepsilon_{2}\right)}^{2}+{\left(\varepsilon_{2}-\varepsilon_{3}\right)}^{2}+{\left(\varepsilon_{3}-\varepsilon_{1}\right)}^{2}\right]}=\sqrt{\frac{2}{3}\left(18\varepsilon^{2}\right)}=2\sqrt{3}\varepsilon$$

For the von-Mises equivalent stress $\bar{\sigma }$ we can again use $\sigma_{1}=\sigma_{2}=\sigma$. The plane stress $\sigma_{3}\approx 0$, so the von-Mises equivalent stress becomes:

(11) $$\bar{\sigma }=\sqrt{\sigma^{2}}=\sigma$$

Therefore

(12) $$\sigma =\bar{\sigma }=K{\bar{\varepsilon }}^{n}=K{\left(2\sqrt{3}\varepsilon \right)}^{n}$$

To determine the strain at which the applied membrane stress equals the material's flow stress we can equate equations (9) and (12) to obtain:

(13) $$\frac{\mathrm{pR}}{2s_{0}}e^{2\varepsilon }=K{\left(2\sqrt{3}\varepsilon \right)}^{n}$$

#### 2.4.2 Bulge test setup and data acquisition

Biaxial bulge tests were performed using an in-house developed experimental setup based on the pneumatic bulge test principle. At least 2 samples were tested per film type. Film samples were cut into circular specimens (37 mm diameter) and mounted onto the pressure chamber using a clamping ring fastened with 8 screws (Fig. 2). During preliminary experiments, samples were marked with concentric circles to monitor possible slippage. Torque applied to clamping screws was increased between successive trials until slippage was no longer observed at an applied torque of 0.4 Nm per screw. This torque was subsequently applied during all experiments and it was assumed that the films did not slip during the biaxial bulge test. During testing, the air pressure of chamber was continuously increased at a rate of 0.04 bar/s until the sample burst, taking 2-4 min depending on sample type. This slow testing velocity was chosen to prevent strain-rate dependent deviations between measurements. The pressure was monitored via a set of an inlet pressure controller (Bronkhorst, IN-PRESS P-5X2CI + F-0XXAI) and an outlet pressure controller (Bronkhorst, IN-PRESS F-0XXAI + P-5X2CI). The pressure was digitally controlled and recorded by the Bronkhorst software suite (FlowDDE, FlowView). Videos of bulge test were simultaneously recorded at 25 frames per second using a Sony FDR-AX53 4K video camera.

#### 2.4.3 Data pre-processing and synchronization

During video processing, computer vision algorithms from the OpenCV library were utilised to extract the contour of the bulge. A pixel-to-millimetre conversion factor was established using a still image of a ruler placed on the same plane as the bulge.

To extract the bulge contour, each frame was first converted to greyscale and subjected to a mild Gaussian blur with a kernel size of 5 by 5 pixels to attenuate sharp edge transitions. A binarization filter was then applied with a saturation threshold with a default value of 80, which was adjusted such that the bulge shape was well defined in all frames. These intermediate binary images were subsequently processed using morphological dilation followed by skeletonization, ensuring that the bulge outline was represented as a continuous, single-pixel-wide line (Fig. 3A). The detected bulge outline was fitted to an ellipse (Fig. 3B) using a least-squares fit.

(14) $$RMSE=\frac{\sum_{i=1}^{N}{\left(d_{i}\right)}^{2}}{N}$$

With N the number of data points used to fit each frame, and $d_{i}$ the shortest distance from the *i*-th data point to the fitted ellipse (Min et al., 2017).

The parameters derived from the fitting procedure include the coordinates of the ellipse centre, the bulge height *h*_*b*_ relative to the fixation point, and the lengths of the semi-major and semi-minor axes *a* and *b* (Fig. 3B). The bulge outline was processed frame by frame to extract the deformation over time. The deformation data were synchronised with the pressure data (Fig. 3C) and saved for subsequent calculations. Data was processed using a custom-written python script using the OpenCV, skimage and numpy packages (Supplementary Material).

#### 2.4.4 Data analysis

Prior to tensile testing, the sample thickness ($s_{0}$) was measured at three evenly distributed locations over the film surface using a digital calliper (Mitutoyo Manufacturing Co. Tld., Japan). Since the film thickness during bulging could not be determined experimentally, a residual function was derived from equation 13:

(15) $$f\left(\varepsilon \right)=\frac{\mathrm{pR}}{2s_{0}}e^{2\varepsilon }-K{\left(2\sqrt{3}\varepsilon \right)}^{n}$$

This residual function was solved numerically to find the strain at which the applied membrane stress equals the material's flow stress. This equilibrium strain was then used to calculate the film thickness and stress at the apex using equations (8) and (5) respectively. The numerical solver employed adaptive bracketing followed by Brent's root-finding method. By repeating this analysis for each frame, true strain-true stress curves were obtained (Fig. 3D).

#### 2.4.5 Statistical analysis

Statistical analysis was performed using the numpy (v2.2.4) and scipy (v1.15.2) packages in Python (v3.13, Python Software Foundation, USA).

## 3 Results and discussion

### 3.1 Formation of crosslinked and interpenetrating networks

To confirm the effectiveness of transglutaminase-induced crosslinking in GC samples, a GC10-reference sample was prepared for which the crosslinking step was omitted. This sample had poor mechanical properties compared to the GC10 sample, confirming that the crosslinking step improved the mechanical integrity (data not shown). Similarly, a AA10-reference sample was prepared in which the formation of the IPN through steeping in CaCl_2_ was omitted. The AA10 sample had significantly improved mechanical properties and thus it is evident that an IPN is indeed formed. No directionality was observed in IPN samples when studied under a polarizing optical microscope (Zeiss AxioScope 5, Germany).

### 3.2 Uniaxial tensile properties

#### 3.2.1 Elasticity

When a stress is applied to a material, it initially deforms elastically. Elastic deformation is reversible and characterized by a linear relationship between applied stress and observed strain. A material's elasticity is quantified by its elastic modulus, or Young's modulus, which is defined as the slope of the stress-strain curve within the elastic regime.

**Fig. 4:**
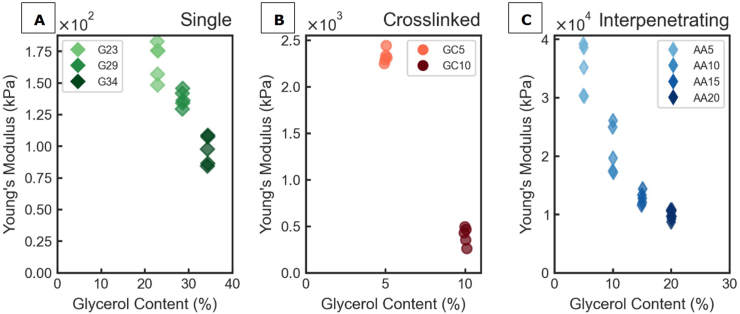
Young's modulus as function of glycerol content for single network films (A), crosslinked network films (B) and interpenetrating network films (C) obtained from uniaxial tensile tests: results of technical replicates are shown.

SN films had the lowest Young's modulus among the tested films, ranging from 85 to 180 kPa (Fig. 4A). Baumgartner et al. (2020) investigated SN gelatine hydrogels similar to those prepared for this study and reported Young's modulus values of 30-300 kPa, depending on the gelatine content. Young's moduli of other SN films made with gelatine, agarose or non-edible polymers were in the range of 12-500 kPa (Hur et al., 2014; Smith et al., 2024; Sun et al., 2024; Suo et al., 2018). SN films made with gelatine are physically crosslinked by triple helical junction zones, with hydrogen bonding, ionic bonds and hydrophobic interactions between polymer chains but lacking stronger bonds such as covalent bonds (Poveda-Reyes et al., 2015; Zhang et al., 2019). In general, polymers in SN are weakly associated and thus do not provide much resistance to elongation (Clark, 1996). This is evident in the small Young's moduli of SN films.

Due to poor mechanical integrity, the GC15 films could not be analysed. Young's moduli of other CN films were an order of magnitude higher than those of SN films, ranging from 260 to 2400 kPa (Fig. 4B). For CN films based on non-edible polymers, Young's moduli between 122 and 5310 kPa have been reported, which matches well with our observations (Lin et al., 2015; Liu et al., 2018; Zheng et al., 2020; Zhu et al., 2018). As polymer chains are covalently crosslinked in CN films, more resistance to uniaxial elongation is expected compared to SN films. In our CN films, covalent bonds between proteins, peptides and primary amines were introduced through the transglutaminase-catalysed formation of ϵ-(γ-glutamyl)-lysine crosslinks within and in between gelatine and casein proteins via an acyl transfer reaction (Chambi and Grosso, 2006; Yildirim and Hettiarachchy, 1998).

The Young's moduli of IPN films were an order of magnitude higher than CN films, ranging between 11600 and 39000 kPa (Fig. 4C). The literature does not report on any edible IPN films, but a broad range of Young's modulus values for non-edible IPN films has been reported, ranging from 1171 kPa to 19000 kPa (Myung et al., 2007; Panteli and Patrickios, 2019; Zhou et al., 2020). In IPN films, two independent polymer networks are intertwined which increases the overall resistance to stretching compared to SN and CN films (Niu et al., 2019; Panteli and Patrickios, 2019; Suo et al., 2018). The crosslink density of the primary network governs the available free volume and the strain dissipation capacity for the secondary network (Lin et al., 2019; Suo et al., 2018), which explains why a wide range of Young's moduli is reported in literature.

Despite the differences in Young's modulus at low plasticizer content of SN, CN and IPN films, increasing plasticizer content always led to a strong reduction in Young's modulus across all film types, making films more flexible and less stiff (Fig. 4). This finding is consistent with reported reductions in Young's modulus for plasticized films composed of zein, alginate, pea protein and various synthetic plastics (Choi and Han, 2001; Gao et al., 2017; Lawton, 2004; Sears and Darby, 1982). Liu et al. and Carvalho reported a negative linear correlation between Young's modulus and the glycerol content of gelatine films (de Carvalho and Grosso, 2004; Liu et al., 2017). Both studies reported a similar effect after inducing crosslinking using transglutaminase, thus matching our observation for SN and CN films. A decrease in Young's modulus as result of plasticizer addition has also been reported for IPNs based on acrylic elastomers (Zhang et al., 2010).

The effects of plasticizers on polymer networks can be attributed to their ability to form hydrogen bonds with polymer chains, replacing intermolecular bonds between polymer chains and increasing the available free volume in the polymer network (Eslami et al., 2023). In the elastic region, over which the Young's modulus is calculated, only reversible deformation occurs. The effect of cross-linkages or interpenetrating networks on deformation behaviour is therefore limited in the elastic region, which explains why the influence of plasticizer content is the same for SN, CN and IPN networks.

#### 3.2.2 Tensile strength

Once a material is stressed beyond its linear elastic regime, it will cross its yield point and enter the plastic regime. Stress applied in the plastic regime changes the microstructure of the material, causing irreversible deformation that persists after removal of the load. Upon further tensile deformation a material will eventually fracture. The strain and stress at the point at this breaking point define a materials tensile strength.

**Fig. 5:**
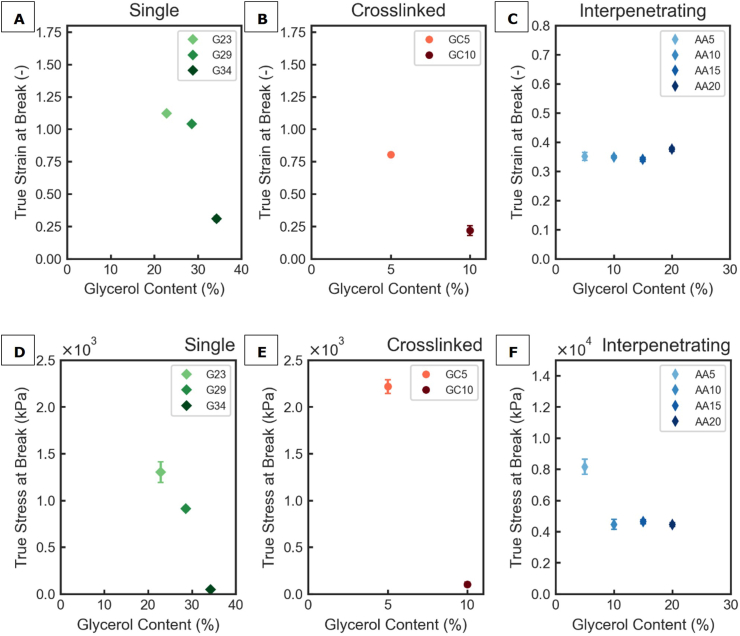
Film strength determined by uniaxial tensile tests: True strain at break as function of glycerol content for gelatine films (A), gelatine-caseinate films (B) and agar-alginate films (C); True stress at break as function of glycerol content for gelatine films (D), gelatine-caseinate films (E) and agar-alginate films (F); test results of at least 5 technical replicates are presented as mean ± SD (standard deviation).

The uniaxial tensile tests revealed the fracture behaviour of the SN, CN and IPN films. The true stress at break ranged from 47 to 1300 for SN films, to 95-2200 for CN films and 4400-8100 kPa for IPN films, depending on plasticizer content (Fig. 5). For gelatine and polyacrylamide SN films, true stress at break values of 18-406 kPa were reported which aligns with our observations (Myung et al., 2007; Suo et al., 2018). For CN films, true stress at break values of 4500-25000 kPa have been reported, which is much higher than for the GC5 and GC10 films (Chambi and Grosso, 2006; Lin et al., 2015; Zhao et al., 2024). Finally, IPN films are reported to have stress at break values of 5000-40000 kPa, with the lower end of that range matching our observations (Hwang et al., 2024; Xu et al., 2021; Yan et al., 2017; Yang et al., 2017; Zhou et al., 2020).

IPN films can withstand much higher stresses before failing compared to SN and CN films. As IPNs consist of a primary polymer network that is covalently crosslinked and a secondary polymer network that is ionically (i.e., physically) crosslinked, stresses on IPN films are distributed over two networks. Stresses can be dissipated by fission of the ionic crosslinks, while the covalent crosslinks remain intact (Bootsma et al., 2017; Sun et al., 2012). At larger deformations, this also prevents cracks from propagating into large ruptures (Chen et al., 2014).

Plasticization of SN and CN films with glycerol resulted in decreased fracture strain and stress (Fig. 5A and B). In contrast, the fracture strain of the alginate-agar IPN film remained unaffected by glycerol addition, and its fracture stress was only reduced between 5% and 10% w/w glycerol content, with no further change observed upon additional plasticizer incorporation (Fig. 5C). A reduction in fracture stress with increasing plasticizer content has been reported for zein, alginate film and pea protein films, but contrary to our observations this is most often accompanied by an increase in fracture strain (Chambi and Grosso, 2006; Choi and Han, 2001; Gao et al., 2017; Lai and Padua, 1997; Lawton, 2004).

A reduction in the strain at break with increasing plasticizer content could be caused by segregation of plasticizer during the drying of the films or incompatibility of the plasticizer and polymer (Gao et al., 2017; Rusli, 2017). Gelatin, agar and alginate have good compatibility with glycerol at relatively lower plasticizer concentrations (Basu et al., 2022; Bhatia et al., 2024; Rusli, 2017). However, when the plasticizer concentration becomes very high, it is likely that phase segregation occurs during drying of cast films. Phase segregation creates regions in the film filled only or mostly by plasticizer, forming pores from which cracks may propagate when the film is stressed (Gao et al., 2017). This could explain the observed reduction in strain at break with increasing levels of plasticizer in SN films in this study (Fig. 5). Furthermore, measured strain at break for CN films was reduced with higher glycerol content. Both GC5 and GC10 possess poor mechanical strength compared to literature reports for CN films, while GC15 was too fragile to test for its mechanical properties. It has been reported in literature that casein has low compatibility with glycerol and has been shown to phase-separate during drying, resulting in distinct regions composed rich in glycerol (Chambi and Grosso, 2006). Such regions can create pores in the film, weakening its mechanical strength. We even observed formation of some small droplets on the surface of GC15 films after drying, which is indicative of phase separation. Finally, Rusli et al. investigated SN films with 1-3% agar and 5-15% glycerol and found that the glycerol concentration did not significantly affect fracture strain or fracture stress during uniaxial extension. A similar trend can be observed for the agar-alginate IPN (Fig. 5). The fracture behaviour of the IPN under uniaxial extension therefore appears to be governed by the stiff agar network, with minimal contribution from the more ductile alginate network.

#### 3.2.3 Strain hardening

Strain hardening (*n*) is a measure of the (nonlinear), often irreversible increase in stress required to sustain tensile deformation. The stress-strain curves of all film types showed clear evidence of plastic deformation at higher strain levels, and thus strain hardening behaviour was expected for all film types. Consistent with this, the plastic region of the stress-strain curves was well captured by the Hollomon law (equation 4), yielding coefficients of determination of R^2^ ≥ 0.98 for all samples.

Increasing the plasticizer content of SN films resulted in a reduction of the strain hardening exponent (Table 2). Plasticizers increase the chain mobility and facilitate distribution of the deformation over a larger fraction of all chains, enhancing the compliance to stretching.

**Table 2:** Strain hardening parameters of single network (SN), crosslinked network (CN), and interpenetrating network (IPN) films with different plasticizer levels.

Film type	K (MPa)	n (−)
**SN: Gelatine**		
**G23**	0.138 ± 0.01	1.43 ± 0.08
**G29**	0.144 ± 0.00	1.25 ± 0.03
**G34**	0.034 ± 0.00	0.27 ± 0.04

**CN: Gelatine-Caseinate**		
**GC5**	0.854 ± 0.02	0.39 ± 0.01
**GC10**	0.311 ± 0.03	0.70 ± 0.05

**IPN: Alginate-agar**		
**AA5**	9.23 ± 0.55	0.57 ± 0.02
**AA10**	4.97 ± 0.69	0.53 ± 0.03
**AA15**	5.70 ± 0.14	0.61 ± 0.00
**AA20**	5.08 ± 0.20	0.66 ± 0.01

For IPN films a slight increase of the strain hardening coefficient was observed with increasing plasticizer content (Table 2). The strain hardening coefficient of acetylated chitosan hydrogels crosslinked with genipin was reported to depend on the balance between weak physical entanglements and strong covalent crosslinks, which could be modulated through the gelation temperature (Mio et al., 2022). We expect that the plasticizers have a similar modulating effect. In our IPN film, the first network is a stiff agar network, whereas the second network is a ductile alginate network. We hypothesize that addition of plasticizer mainly influences the second, more ductile network which affects its energy dissipating capacity. This could have resulted in the observed increased strain hardening coefficient of the IPN with the increase of glycerol concentrations.

### 3.3 Biaxial tensile properties

#### 3.3.1 Bulge shape fitting

**Fig. 6:**
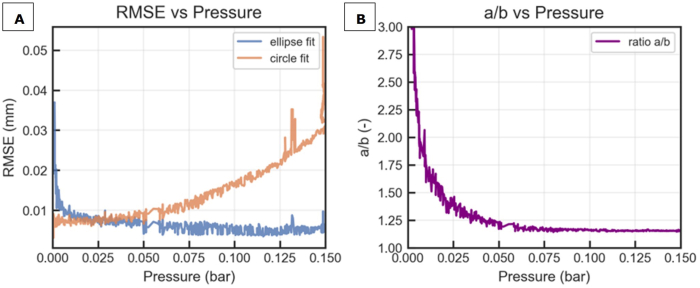
A) Absolute root mean square error (RMSE) of a circle and ellipse fitted to the bulge edge as function of pressure for sample G34; B) the ratio of the length of the major axis-a over the minor axis-b for an ellipse fitted to the bulge edge as function of pressure for sample G34.

For biaxial extensional testing, the bulge test as described before was used. During preliminary testing the approximation of the bulge outline with a circle and ellipse were compared using a representative SN film (G34). Fitting with a circle introduced a systematic error which increases as the bulge becomes larger with increasing pressure (as the deformation of the film becomes larger, Fig. 6A). The ratio of the major to minor axis of the fitted ellipse never approaches 1 (Fig. 6B), a ratio which indicates a circle as the major and minor axis of a circle are of equal length, thus $\frac{a}{b}=1$. Fitting the bulge outline with an ellipse provided a much lower RMSE over the full pressure range (equation 14). This is in agreement with Mulder et al. (2015) who reported that a spheroid (an axisymmetric ellipse) provides a superior fit to a sphere (an axisymmetric circle) during bulge testing of metals. Accordingly, we used an ellipse shape to fit the bulge surface in this work.

#### 3.3.2 Biaxial tensile strength and comparison with uniaxial tensile strength

**Fig. 7:**
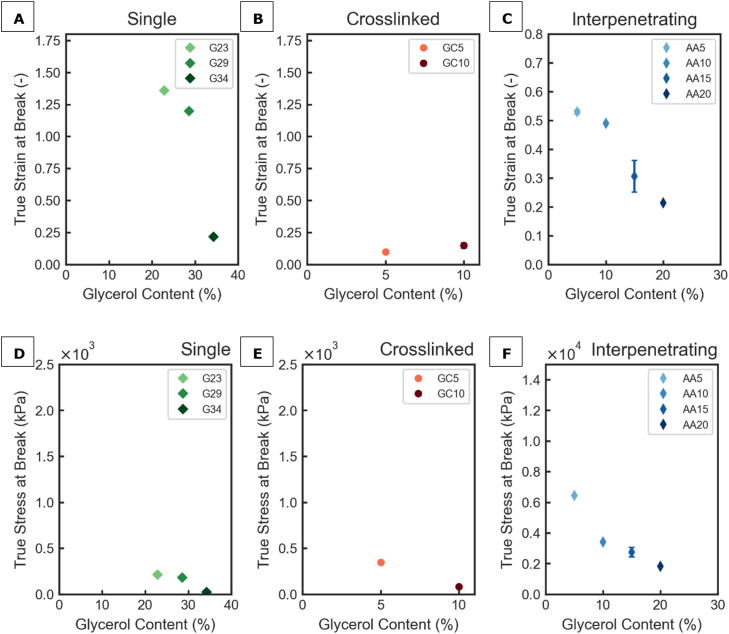
Film strength determined by biaxial tensile tests: True strain at break as function of glycerol content for gelatine films (A), gelatine-caseinate films (B) and agar-alginate films (C). True stress at break as function of glycerol content for gelatine films (D), gelatine-caseinate films (E) and agar-alginate films (F); test results of at least technical replicates were shown as mean ± SD (standard deviation).

The biaxial tensile tests revealed the biaxial fracture behaviour of the SN, CN and IPN films as affected by glycerol concentrations (Fig. 7). The true stress at break determined by biaxial tensile tests ranged from 23 to 210, to 81-345 and 1800-6400 kPa for SN, CN films and IPN films, respectively, depending on plasticizer content (Fig. 7). Full true strain-true stress curves can be found in the section ‘Biaxial strain stress curves’ of the Supplementary Materials. Interestingly, unlike the uniaxial test result which indicated that the plasticizer content did not affect the true strain and stress at break for IPN films (Fig. 5C and F), the biaxial test results clearly indicate that the true biaxial strain and stress at break decrease with plasticizer content (Fig. 7C and F). The decrease in biaxial fracture strain and stress can possibly be explained by the increased mobility of the chains due to the plasticizer, resulting in creep and ultimately allowing failure of the film under stress. Although same film samples were used for biaxial and uniaxial tensile tests, distinct results were shown. This highlights the necessity of performing both tests to obtain more representative information on how the films would perform when used in different applications where uniaxial/biaxial stresses are relevant.

**Fig. 8:**
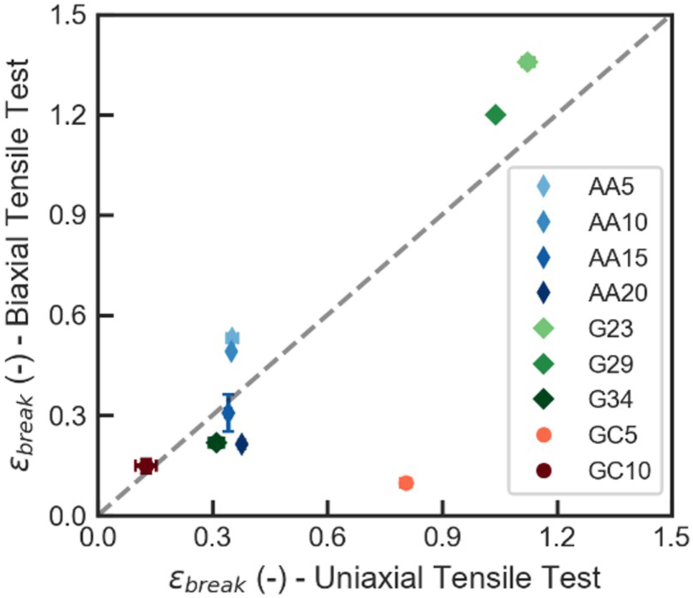
True strain at break *ε*_*break*_ for SN films (G), CN films (GC) and IPN films (AA) compared between uniaxial tensile test and biaxial tensile test.

In general, true strain and stress at break values obtained via this biaxial test were of a similar order of magnitude to those determined through uniaxial tensile testing (Fig. 8), supporting the validity of the test results. Interestingly, a much lower fracture strain was observed for GC5 film during biaxial test, as compared to uniaxial test result (Fig. 8). During the test, it was noticed that the GC5 films were somewhat brittle. When the film was fixated onto the setup, small surface defects were caused by manipulation of the film. These defects could easily propagate, causing sudden and large ruptures during inflation, which is also reflected in the lower fracture stress of this sample during biaxial test (Fig. 7E), as compared to uniaxial test (Fig. 5E).

Additionally, the computer vision based bulge test enables repeated stretching of films using an oscillatory pressure function, which could be used to test fatigue properties of thin films. While an in-depth investigation of this was out of scope for the current study, preliminary results can be found in the section ‘Fatigue testing using biaxial tensile test setup’ of the Supplementary Materials.

## 4 Conclusions

The mechanical properties of different types of edible biopolymer films as affected by their plasticizer content were investigated. Single-network (SN), crosslinked-network (CN) and interpenetrating-network (IPN) films consisting of gelatine, and the combinations gelatine-caseinate and alginate-agar, respectively, were characterized by combining a standard uniaxial tensile test and an in-house developed biaxial bulge test.

Glycerol as plasticizer increased the film elasticity for all film types but reduced their stress at break. Contrary to literature, glycerol addition was found to reduce strain at break, which could be related to segregation of plasticizer during the drying of the films. While uniaxial deformation showed no effects of the plasticizer content on strain and stress at break with IPN films, biaxial testing revealed reductions in tensile strength. We attribute this to a reduction of the energy dissipating capacity of the secondary network in the interpenetrating-network film during biaxial extension. Although the interpenetrating networks create strong films with better properties than their single network counterparts, future studies should focus on improving their biaxial extensibility further and assessing the effects of successive load cycles on tensile properties.

The starkly differing results between uniaxial and biaxial deformation for IPN films indicate that it is important to choose a deformation assessment that is relevant to the application. Moreover, this indicates that different network types respond differently to uniaxial and biaxial deformation, which is likely related to the mechanisms by which they dissipate stresses. In many practical applications, deformation of films is biaxial, and hence it is important to use a biaxial test method. Since data extraction with our custom biaxial tensile test is simple and fast, it thus represents an improvement in both methodology and in convenience compared to existing biaxial tensile tests.

## CRediT authorship contribution statement

**Quinten Steffens:** Conceptualization, Data curation, Formal analysis (lead), Investigation, Methodology, Software, Supervision, Visualization, Writing - original draft, review & editing. **Dyllan Gan Yu-Jing:** Data curation, Formal analysis, Methodology, Software, Investigation. **Naomi Schuppert:** Data curation, Formal analysis, Methodology, Software, Investigation. **Ruud van der Sman:** Conceptualization, Formal analysis, Methodology, Writing - review & editing, Supervision. **Remko Boom:** Conceptualization, Formal analysis, Funding acquisition, Methodology, Writing - review & editing, Supervision. **Yizhou Ma:** Writing - review & editing, Formal analysis. **Lu Zhang:** Conceptualization, Formal analysis, Methodology, Supervision, Writing - review & editing.

## Statement of AI usage

Claude-ai (Sonnet 3.5) was used to improve the python scripts used for the computer-vision analysis. After using this tool, the authors reviewed and edited the code as needed and they take full responsibility for the final version of the script (see Supplementary Material).

## Declaration of competing interest

The authors declare that they have no known competing financial interests or personal relationships that could have appeared to influence the work reported in this paper.

## Data Availability

Data will be made available on request.

## References

[bib1] Basu T., Bhutani U., Majumdar S. (2022). Cross-linker-free sodium alginate and gelatin hydrogels: a multiscale biomaterial design framework. J. Mater. Chem. B.

[bib2] Baumgartner M., Hartmann F., Drack M., Preninger D., Wirthl D., Gerstmayr R., Lehner L., Mao G., Pruckner R., Demchyshyn S., Reiter L., Strobel M., Stockinger T., Schiller D., Kimeswenger S., Greibich F., Buchberger G., Bradt E., Hild S., Bauer S., Kaltenbrunner M. (2020). Resilient yet entirely degradable gelatin-based biogels for soft robots and electronics. Nat. Mater..

[bib3] Bhatia S., Al-Harrasi A., Almohana I.H., Albayati M.S., Jawad M., Shah Y.A., Ullah S., Philip A.K., Halim S.A., Khan A., Anwer M.K., Koca E., Aydemir L.Y., Dıblan S. (2024). The physicochemical properties and molecular docking study of plasticized amphotericin B loaded sodium alginate, carboxymethyl cellulose, and gelatin-based films. Heliyon.

[bib4] Bootsma K., Fitzgerald M.M., Free B., Dimbath E., Conjerti J., Reese G., Konkolewicz D., Berberich J.A., Sparks J.L. (2017). 3D printing of an interpenetrating network hydrogel material with tunable viscoelastic properties. J. Mech. Behav. Biomed. Mater..

[bib5] Brindle L.P., Krochta J.M. (2008). Physical properties of whey protein–hydroxypropylmethylcellulose blend edible films. J. Food Sci..

[bib6] Chambi H., Grosso C. (2006). Edible films produced with gelatin and casein cross-linked with transglutaminase. Food Res. Int..

[bib7] Charalambides M., Wanigasooriya L., Williams G., Chakrabarti S. (2002). Biaxial deformation of dough using the bubble inflation technique. I. Experimental. Rheologica Acta.

[bib8] Chen Q., Zhu L., Huang L., Chen H., Xu K., Tan Y., Wang P., Zheng J. (2014). Fracture of the physically cross-linked first network in hybrid double network hydrogels. Macromolecules.

[bib9] Choi W.-S., Han J.H. (2001). Physical and mechanical properties of pea-protein-based edible films. J. Food Sci..

[bib10] Clark A.H. (1996). Biopolymer gels. Curr. Opin. Colloid Interface Sci..

[bib11] de Carvalho R.A., Grosso C.R.F. (2004). Characterization of gelatin based films modified with transglutaminase, glyoxal and formaldehyde. Food Hydrocoll..

[bib12] Dudderar T.D., Koch F.B., Doerries E.M. (1977). Measurement of the shapes of foil bulge-test samples: post-test shapes of bulge samples are determined by optical contouring. Radius-of-curvature distributions are evaluated using computer-based polynomial-spline fitting routines to analyze the resulting interferometric-fringe data. Exp. Mech..

[bib13] Dufour M., Chaunier L., Hugon F., Dugué A., Kansou K., Saulnier L., Della Valle G. (2024). From alveograph test to extensional behavior of wheat flour dough. Rheol. Acta.

[bib14] Ebrahimi R., Lotfabad F.R. (2022). A comprehensive mathematical analysis on achieving stress–strain behavior at large strains in bulge test. Iran J Sci Technol Trans Mech Eng.

[bib15] Edward S., Golecki H.M. (2023). Gelatin soft actuators: benefits and opportunities. Actuators.

[bib16] Eslami Z., Elkoun S., Robert M., Adjallé K. (2023). A review of the effect of plasticizers on the physical and mechanical properties of alginate-based films. Molecules.

[bib17] Gao C., Pollet E., Avérous L. (2017). Properties of glycerol-plasticized alginate films obtained by thermo-mechanical mixing. Food Hydrocoll..

[bib18] Güner A., Brosius A., Tekkaya A.E. (2009). Analysis of the hydraulic bulge test with FEA concerning the accuracy of the determined flow curves. Key Eng. Mater..

[bib19] Guo J., Liu Y.-C., Yang X.-Q., Jin Y.-C., Yu S.-J., Wang J.-M., Hou J.-J., Yin S.-W. (2014). Fabrication of edible gellan gum/soy protein ionic-covalent entanglement gels with diverse mechanical and oral processing properties. Food Res. Int..

[bib20] Hallfeldt T., Hotz W., Leppin C., Keller S., Friebe H., Till E.T., Müller R., Vučetić M., Vegter H. (2014). Comprehensive Materials Processing.

[bib21] Hartmann F., Baumgartner M., Kaltenbrunner M. (2021). Becoming sustainable, the new frontier in soft robotics. Adv. Mater..

[bib22] Hill R. (1950). C. A theory of the plastic bulging of a metal diaphragm by lateral pressure. London, Edinburgh Dublin Phil. Mag. J. Sci..

[bib23] Huang C.K., Lou W.M., Tsai C.J., Wu T.-C., Lin H.-Y. (2007). Mechanical properties of polymer thin film measured by the bulge test. Thin Solid Films.

[bib24] Hur J., Im K., Kim S.W., Kim J., Chung D.-Y., Kim T.-H., Jo K.H., Hahn J.H., Bao Z., Hwang S., Park N. (2014). Polypyrrole/agarose-based electronically conductive and reversibly restorable hydrogel. ACS Nano.

[bib25] Hwang U., Moon H., Park J., Jung H.W. (2024). Crosslinking and swelling properties of pH-Responsive Poly(Ethylene Glycol)/Poly(Acrylic acid) interpenetrating polymer network hydrogels. Polymers.

[bib26] Janes M.E., Dai Y. (2012). Advances in Meat, Poultry and Seafood Packaging.

[bib28] Jødal A.-S.S., Larsen K.L. (2021). Investigation of the relationships between the alveograph parameters. Sci. Rep..

[bib27] Jeong D., Kim C., Kim Y., Jung S. (2020). Dual crosslinked carboxymethyl cellulose/polyacrylamide interpenetrating hydrogels with highly enhanced mechanical strength and superabsorbent properties. Eur. Polym. J..

[bib29] Kaya S., Altan T., Groche P., Klöpsch C. (2008). Determination of the flow stress of magnesium AZ31-O sheet at elevated temperatures using the hydraulic bulge test. International Journal of Machine Tools and Manufacture, Advances in Sheet Metal Forming Applications.

[bib30] Khalesi H., Lu W., Nishinari K., Fang Y. (2020). New insights into food hydrogels with reinforced mechanical properties: a review on innovative strategies. Adv. Colloid Interface Sci..

[bib31] Khwaldia K., Banon S., Perez C., Desobry S. (2004). Properties of sodium caseinate film-forming dispersions and films. J. Dairy Sci..

[bib75] Kozempel M., Tomasula P.M. (2004). Development of a continuous process to make casein films. J. Agric. Food Chem..

[bib32] Kruglov A.A., Enikeev F.U., Lutfullin R.Ya (2002). Superplastic forming of a spherical shell out a welded envelope. Mater. Sci. Eng., A.

[bib33] Lai H., Padua G.W. (1997). Properties and microstructure of plasticized zein films. Cereal Chem..

[bib34] Lawton J.W. (2004). Plasticizers for zein: their effect on tensile properties and water absorption of zein films. Cereal Chem..

[bib35] Lazarescu L., Comsa D.S., Banabic D. (2011). Analytical and experimental evaluation of the stress-strain curves of sheet metals by hydraulic bulge tests. KEM.

[bib36] Leceta I., Etxabide A., Cabezudo S., De La Caba K., Guerrero P. (2014). Bio-based films prepared with by-products and wastes: environmental assessment. J. Clean. Prod..

[bib37] Li J., Qiu Y., Wang H., Wang Z. (2019). Estimation of the strength coefficient and strain hardening exponent from monotonic tensile properties of steels. Int J Steel Struct.

[bib38] Li L., Guan S., Yang L., Qin X., Feng W. (2018). Mechanical and adhesive properties of ploy(ethylene glycerol) diacrylate based hydrogels plasticized with PEG and glycerol. Chem. Res. Chin. Univ..

[bib39] Li X., Xu H., Yang Q., Long S., Yuan Y., Chen M. (2018). And Preparation Method Thereof.

[bib40] Lin F., Lu X., Wang Z., Lu Q., Lin G., Huang B., Lu B. (2019). In situ polymerization approach to cellulose–polyacrylamide interpenetrating network hydrogel with high strength and pH-responsive properties. Cellulose.

[bib41] Lin P., Ma S., Wang X., Zhou F. (2015). Molecularly engineered dual-crosslinked hydrogel with ultrahigh mechanical strength, toughness, and good self-recovery. Adv. Mater..

[bib42] Liu F., Chiou B.-S., Avena-Bustillos R.J., Zhang Y., Li Y., McHugh T.H., Zhong F. (2017). Study of combined effects of glycerol and transglutaminase on properties of gelatin films. Food Hydrocoll..

[bib43] Liu S., Zheng R., Chen S., Wu Y., Liu H., Wang P., Deng Z., Liu L. (2018). A compliant, self-adhesive and self-healing wearable hydrogel as epidermal strain sensor. J. Mater. Chem. C.

[bib44] Min J., Stoughton T.B., Carsley J.E., Carlson B.E., Lin J., Gao X. (2017). Accurate characterization of biaxial stress-strain response of sheet metal from bulge testing. Int. J. Plast..

[bib45] Mio L., Sacco P., Donati I. (2022). Influence of temperature and polymer concentration on the nonlinear response of highly acetylated chitosan–genipin hydrogels. Gels.

[bib46] Mulder J., Vegter H., Aretz H., Keller S., Van Den Boogaard A.H. (2015). Accurate determination of flow curves using the bulge test with optical measuring systems. J. Mater. Process. Technol..

[bib47] Mulder J., Vegter H., Aretz H., Van Den Boogaard T. (2013). Accurate evaluation method for the hydraulic bulge test. KEM.

[bib48] Myung D., Koh W., Ko J., Hu Y., Carrasco M., Noolandi J., Ta C.N., Frank C.W. (2007). Biomimetic strain hardening in interpenetrating polymer network hydrogels. Polymer.

[bib49] Nath P.C., Debnath S., Sridhar K., Inbaraj B.S., Nayak P.K., Sharma M. (2022). A comprehensive review of food hydrogels: principles, formation mechanisms, microstructure, and its applications. Gels.

[bib50] Niu Y., Xia Q., Gu M., Yu L.Lucy (2019). Interpenetrating network gels composed of gelatin and soluble dietary fibers from tomato peels. Food Hydrocoll..

[bib51] Panteli P.A., Patrickios C.S. (2019). Multiply interpenetrating polymer networks: preparation, mechanical properties, and applications. Gels.

[bib52] Poveda-Reyes S., Mellera-Oglialoro L.R., Martínez-Haya R., Gamboa-Martínez T.C., Gómez Ribelles J.L., Gallego Ferrer G. (2015). Reinforcing an injectable gelatin hydrogel with PLLA microfibers: two routes for short fiber production. Macromol. Mater. Eng..

[bib53] Rees D.W.A. (1995). Plastic flow in the elliptical bulge test. Int. J. Mech. Sci..

[bib54] Rusli A. (2017). Physical and mechanical properties of agar based edible film with glycerol plasticizer.

[bib55] Sears J.K., Darby J.R. (1982).

[bib76] Shintake J., Sonar H., Piskarev E., Paik J., Floreano D. (2017).

[bib56] Smith A.M., Inocencio D.G., Pardi B.M., Gopinath A., Andresen Eguiluz R.C. (2024). Facile determination of the poisson's ratio and young's modulus of polyacrylamide gels and polydimethylsiloxane. ACS Appl. Polym. Mater..

[bib57] Stachowicz F., Rzeszowska P. (2003). Presented at the 5th International Multidisciplinary Conference.

[bib58] Sun J.-Y., Zhao X., Illeperuma W.R.K., Chaudhuri O., Oh K.H., Mooney D.J., Vlassak J.J., Suo Z. (2012). Highly stretchable and tough hydrogels. Nature.

[bib59] Sun X., Liang H., Ye L. (2024). Strong, tough and conductive single-network hydrogels based on deswelling and the salting-out effect. Soft Matter.

[bib60] Suo H., Zhang D., Yin J., Qian J., Wu Z., Fu J. (2018). Interpenetrating polymer network hydrogels composed of chitosan and photocrosslinkable gelatin with enhanced mechanical properties for tissue engineering. Materials science & engineering. C, Materials for biological applications.

[bib61] Walker S., Rueben J., Volkenburg T.V., Hemleben S., Grimm C., Simonsen J., Mengüç Y. (2017). Using an environmentally benign and degradable elastomer in soft robotics. Int J Intell Robot Appl.

[bib62] Wittaya T. (2012). Structure and Function of Food Engineering.

[bib63] Xu J., Guo Z., Chen Y., Luo Y., Xie S., Zhang Y., Tan H., Xu L., Zheng J. (2021). Tough, adhesive, self-healing, fully physical crosslinked κ-CG-K+/pHEAA double-network ionic conductive hydrogels for wearable sensors. Polymer.

[bib64] Yan X., Chen Q., Zhu L., Chen H., Wei D., Chen F., Tang Z., Yang J., Zheng J. (2017). High strength and self-healable gelatin/polyacrylamide double network hydrogels. J. Mater. Chem. B.

[bib65] Yang F., Tadepalli V., Wiley B.J. (2017). 3D printing of a double network hydrogel with a compression strength and elastic modulus greater than those of cartilage. ACS Biomater. Sci. Eng..

[bib66] Yildirim M., Hettiarachchy N.s. (1998). Properties of films produced by cross-linking whey proteins and 11S globulin using transglutaminase. J. Food Sci..

[bib67] Yin S., Yao D.R., Song Y., Heng W., Ma X., Han H., Gao W. (2024). Wearable and implantable soft robots. Chem. Rev..

[bib68] Yoo S., Krochta J.M. (2011). Whey protein–polysaccharide blended edible film formation and barrier, tensile, thermal and transparency properties. J. Sci. Food Agric..

[bib69] Zhang H., Düring L., Kovacs G., Yuan W., Niu X., Pei Q. (2010). Interpenetrating polymer networks based on acrylic elastomers and plasticizers with improved actuation temperature range. Polym. Int..

[bib70] Zhang L., Liu J., Zheng X., Zhang A., Zhang X., Tang K. (2019). Pullulan dialdehyde crosslinked gelatin hydrogels with high strength for biomedical applications. Carbohydr. Polym..

[bib71] Zhao Z., Fan L., Song G., Huo M. (2024). Micelle-cross-linked hydrogels with strain stiffening properties regulated by intramicellar cross-linking. Chem. Mater..

[bib72] Zheng Q., Zhao L., Wang J., Wang S., Liu Y., Liu X. (2020). High-strength and high-toughness sodium alginate/polyacrylamide double physically crosslinked network hydrogel with superior self-healing and self-recovery properties prepared by a one-pot method. Colloids Surf. A Physicochem. Eng. Asp..

[bib73] Zhou L., Pei X., Fang K., Zhang R., Fu J. (2020). Super tough, ultra-stretchable, and fast recoverable double network hydrogels physically crosslinked by triple non-covalent interactions. Polymer.

[bib74] Zhu F., Lin J., Wu Z.L., Qu S., Yin J., Qian J., Zheng Q. (2018). Tough and conductive hybrid hydrogels enabling facile patterning. ACS Appl. Mater. Interfaces.

