# Supplementary material for: Mechanical characterization of elastic edible films with single, crosslinked and interpenetrating biopolymer networks using combined uniaxial and biaxial analysis
Source: Curr Res Food Sci. 2026 Mar 13;12:101381. doi: 10.1016/j.crfs.2026.101381 (PMC13050118; doi:10.1016/j.crfs.2026.101381)
Supplement: Multimedia component 1 [file mmc1.docx]

**Mechanical characterization of elastic edible films with single, crosslinked and interpenetrating polymer networks using combined uniaxial and biaxial analysis**

Quinten Steffens^a^, Dyllan Gan Yu-Jing^a^, Naomi Schuppert^a^, Ruud van der Sman^a,b^, Remko Boom^a,d^, Yizhou Ma^a,c^, Lu Zhang^a,*^

^a^ Laboratory of Food Process Engineering, Wageningen University & Research,

Bornse Weilanden 9, 6708 WG Wageningen, the Netherlands

[quinten.steffens@wur.nl](mailto:quinten.steffens@wur.nl), [ruud.vandersman@wur.nl](mailto:ruud.vandersman@wur.nl), [remko.boom@wur.nl](mailto:remko.boom@wur.nl), [yizhou.ma@wur.nl](mailto:yizhou.ma@wur.nl), [lu1.zhang@wur.nl](mailto:lu1.zhang@wur.nl)

^b^ Food & Biobased Research, Wageningen University & Research,

Bornse Weilanden 9, 6708 WG Wageningen, the Netherlands

^c^ Laboratory of Food Quality and Design, Wageningen University & Research,

Bornse Weilanden 9, 6708 WG Wageningen, the Netherlands

^d^ Food Science department, University of Copenhagen,
Rolighedsvej 26, 1958 Frederiksberg, Denmark

* Corresponding author. Tel: + 31 (0) 317 485411. Email: [lu1.zhang@wur.nl](mailto:lu1.zhang@wur.nl)

## Python code

Code is provided in a separate pdf. Python version 3.13 was used with the packages listed in Fig. S 1.


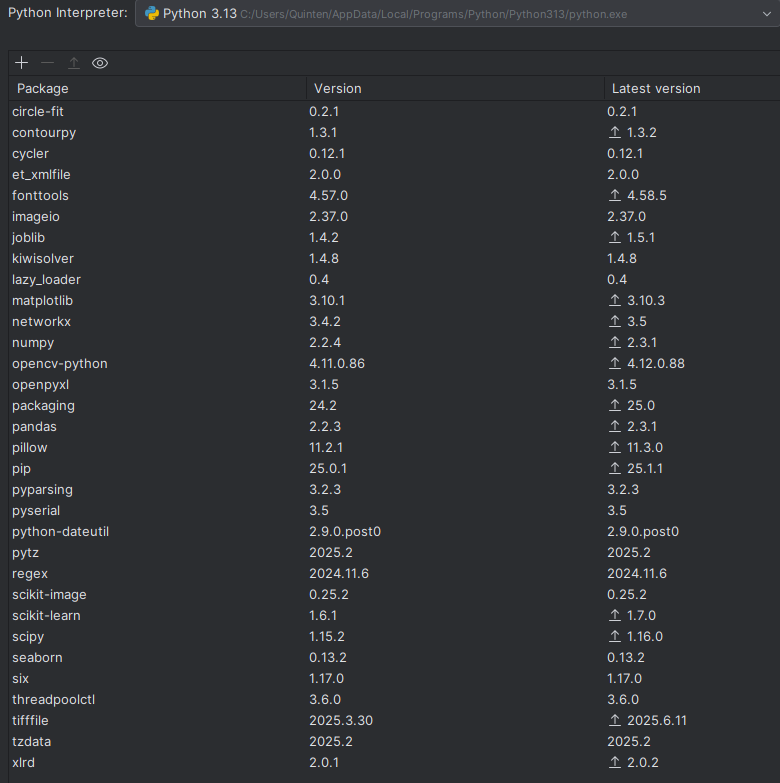


Fig. S 1 Python package

## Film thickness

Table S 1 Average film thickness per sample type

| Sample type | S_0_ (mm) |
| --- | --- |
| AA5 | 0.146±0.002 |
| AA10 | 0.149±0.014 |
| AA15 | 0.256±0.015 |
| AA20 | 0.590±0.056 |
| G23 | 2.173±0.074 |
| G29 | 2.168±0.215 |
| G34 | 1.654±0.399 |
| GC5 | 0.362±0.016 |
| GC10 | 0.347±0.007 |

## Young’s modulus

Table S 2 Statistics related to Young’s moduli obtained from uniaxial tensile testing

| Sample type | Average R² | Average Young's Modulus (kPa) | Std. Dev. (kPa) |
| --- | --- | --- | --- |
| AA5 | 0.9836 | 34731 | 4301 |
| AA10 | 0.9735 | 21067 | 4180 |
| AA15 | 0.9416 | 12832 | 1079 |
| AA20 | 0.9595 | 9963 | 723.5 |
| G23 | 0.9892 | 167.8 | 14.50 |
| G29 | 0.9905 | 139.3 | 43.52 |
| G34 | 0.9762 | 83.97 | 28.89 |
| GC5 | 0.8835 | 2325 | 73.18 |
| GC10 | 0.9649 | 400.92 | 94.46 |

## Biaxial strain stress curves

Strain stress curves obtained by bulge testing.


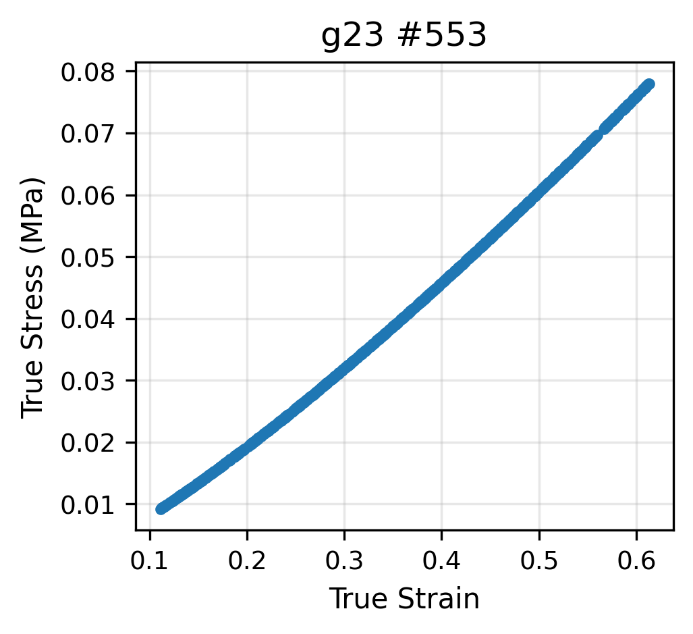

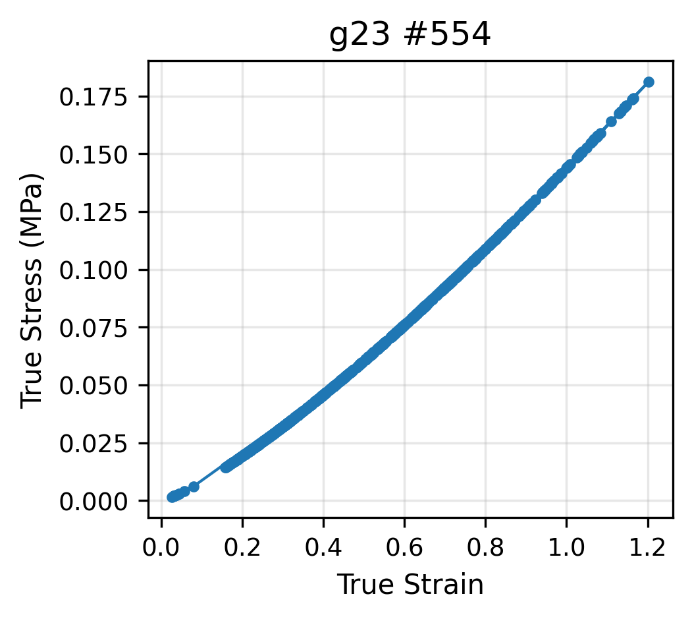


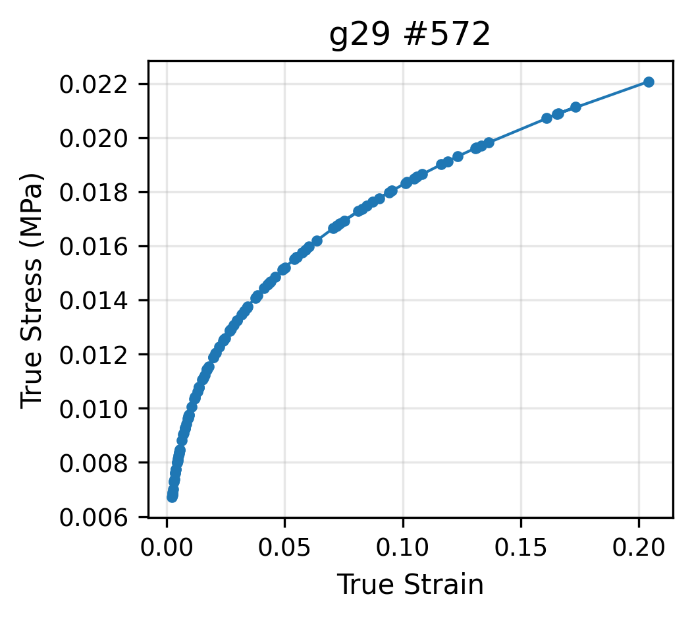

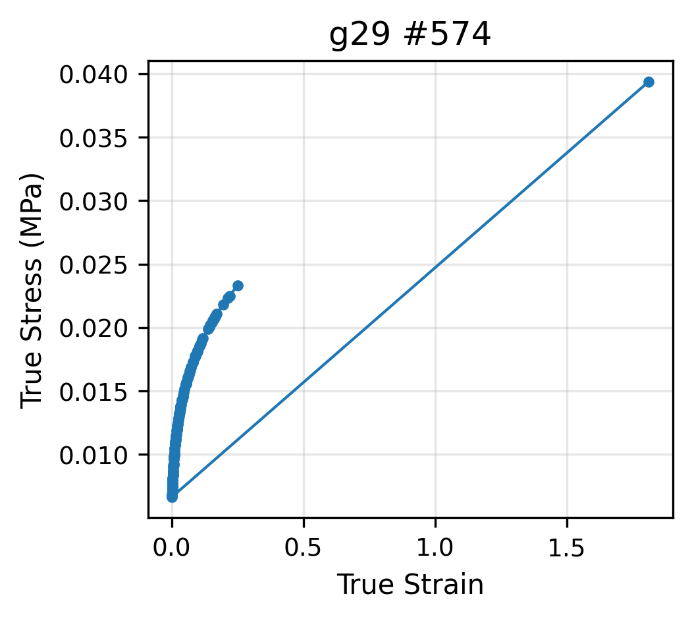


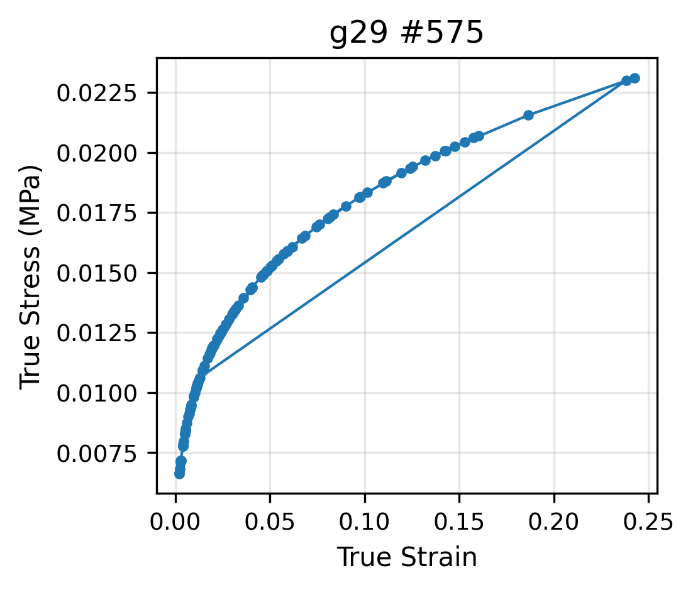

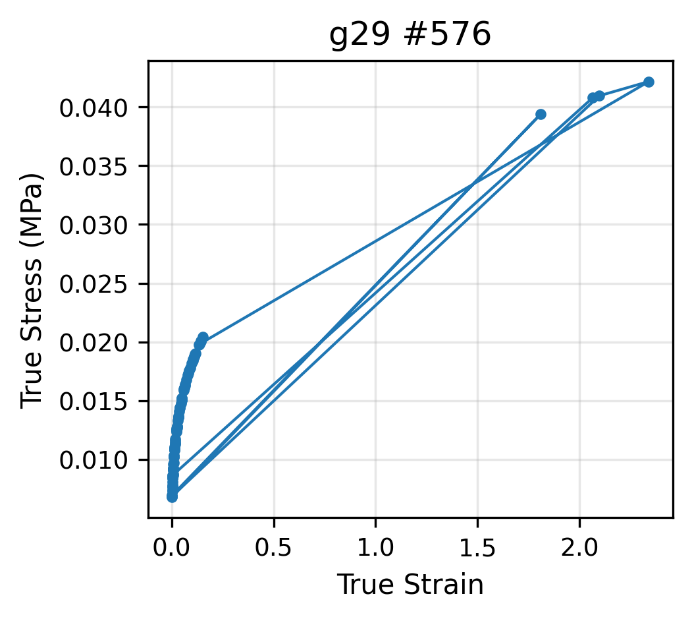


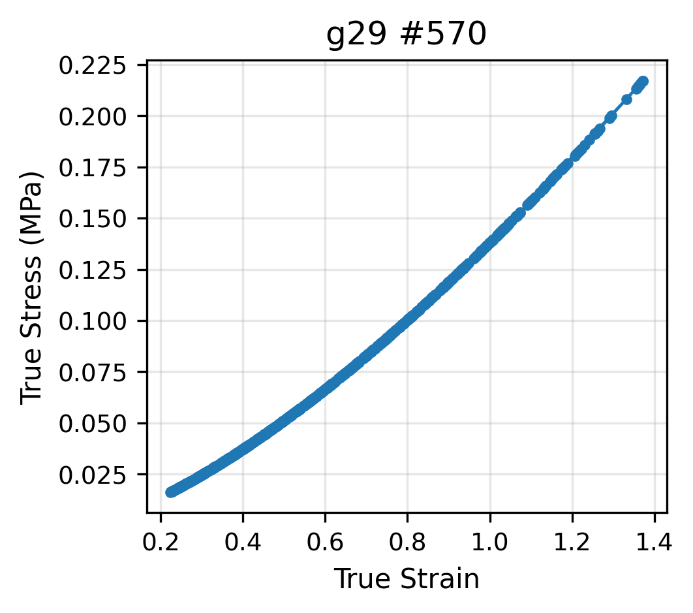

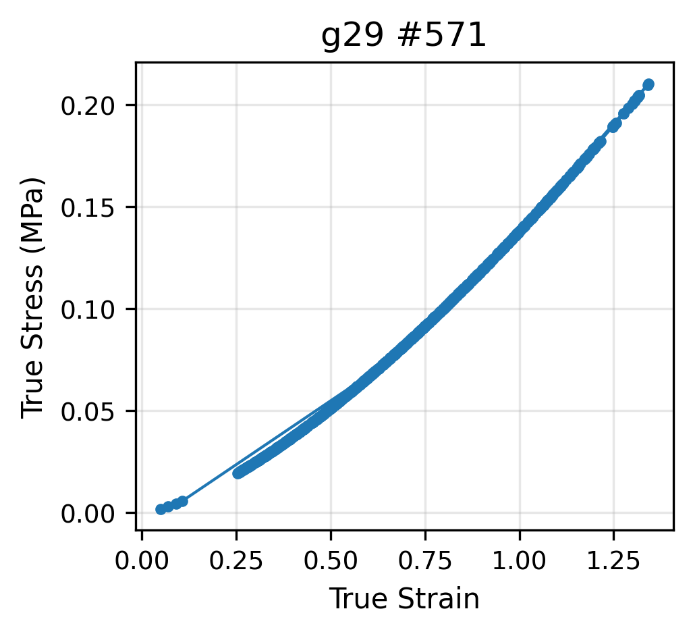


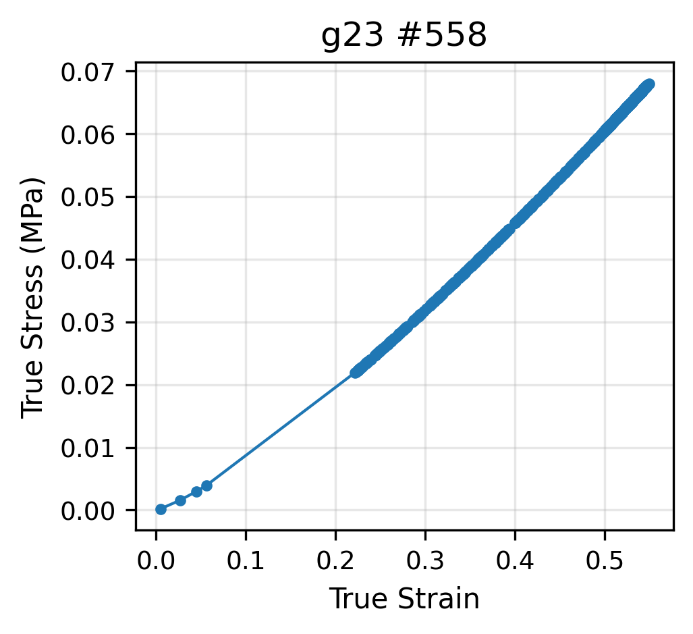

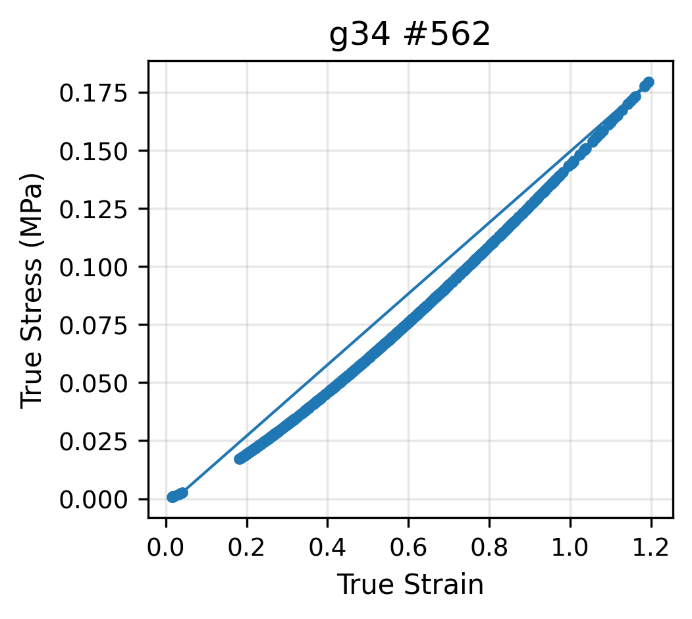


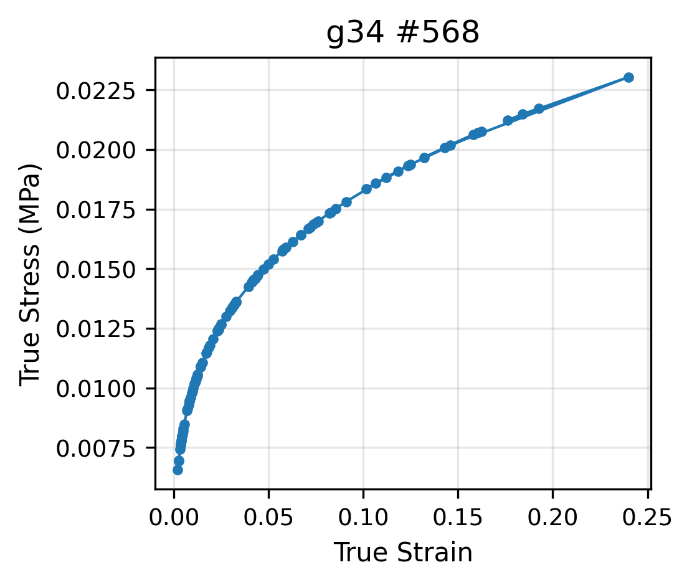

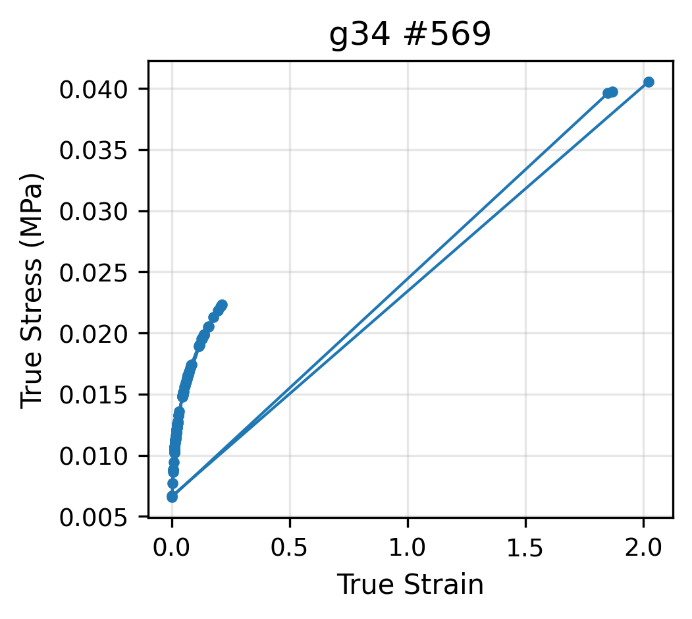


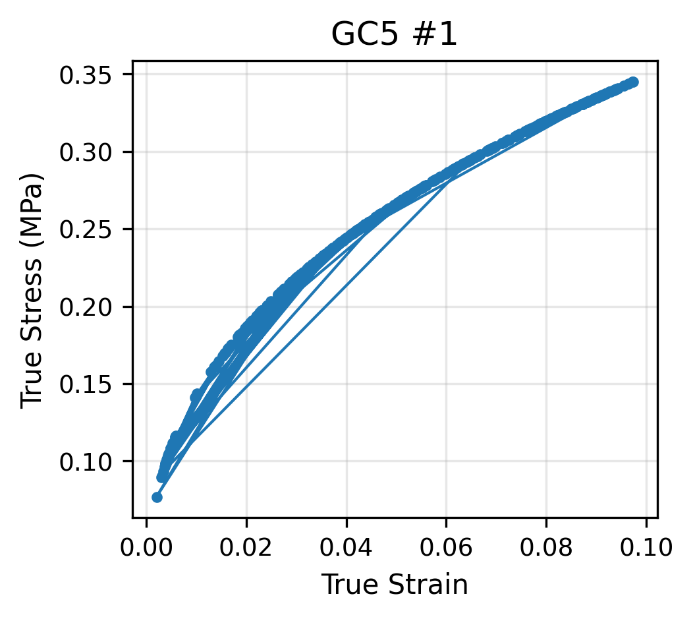

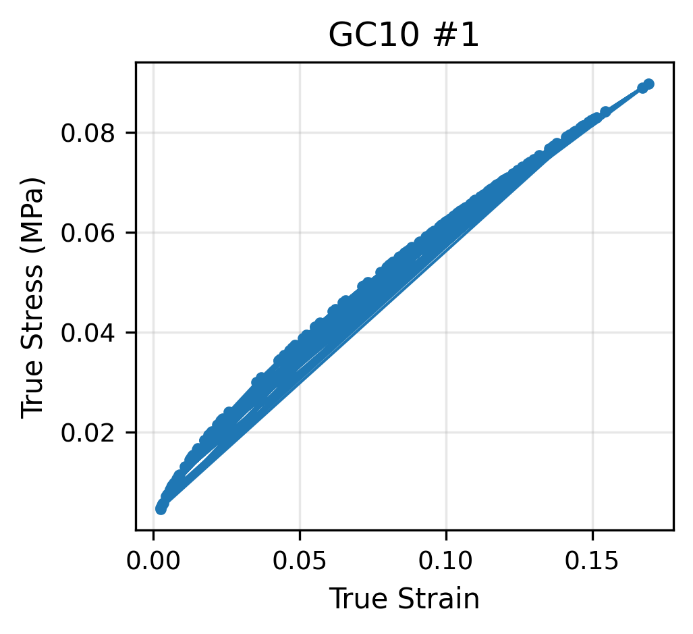


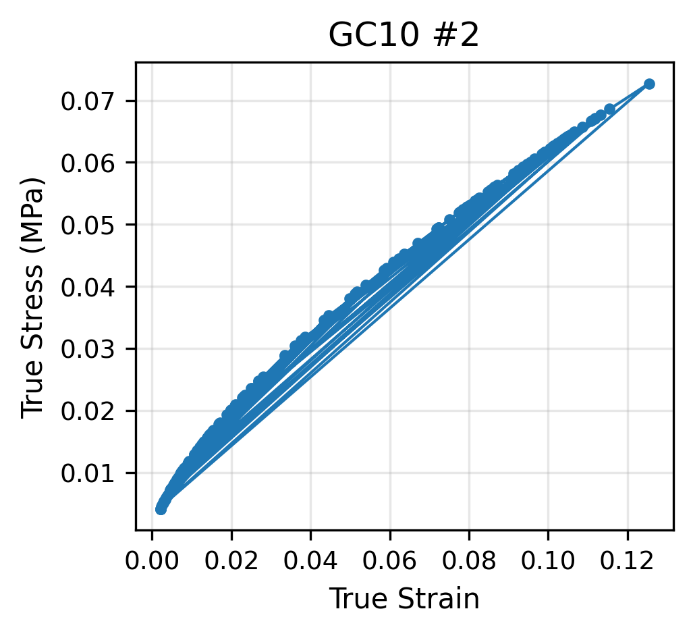

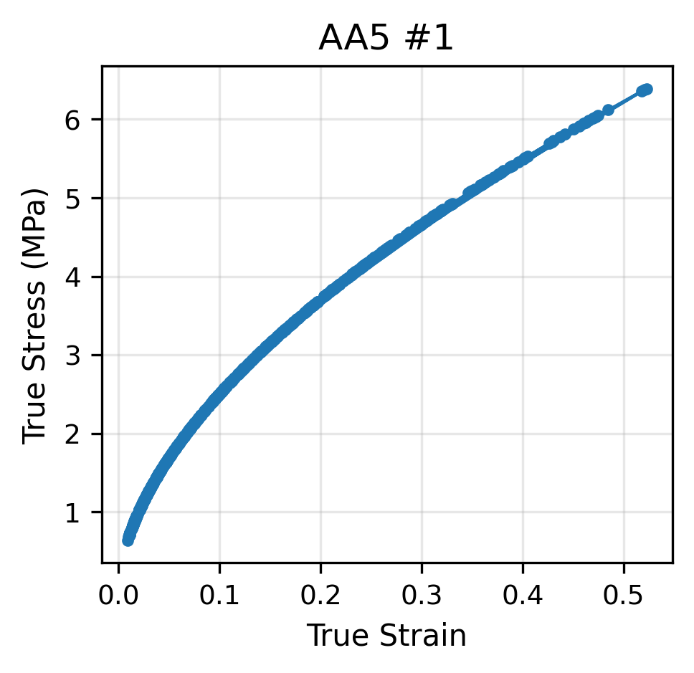


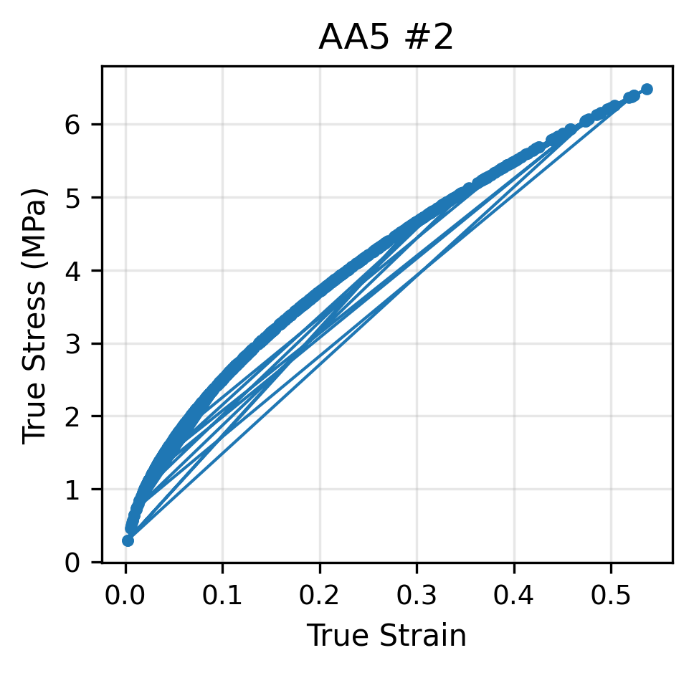

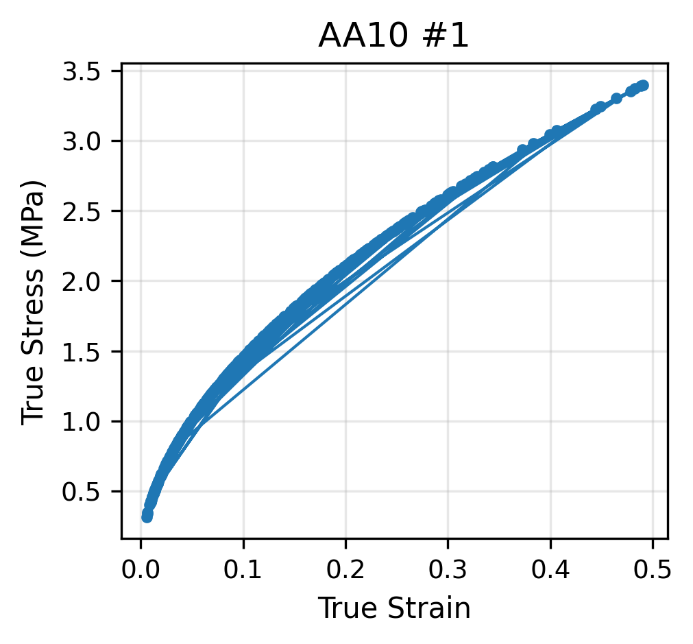


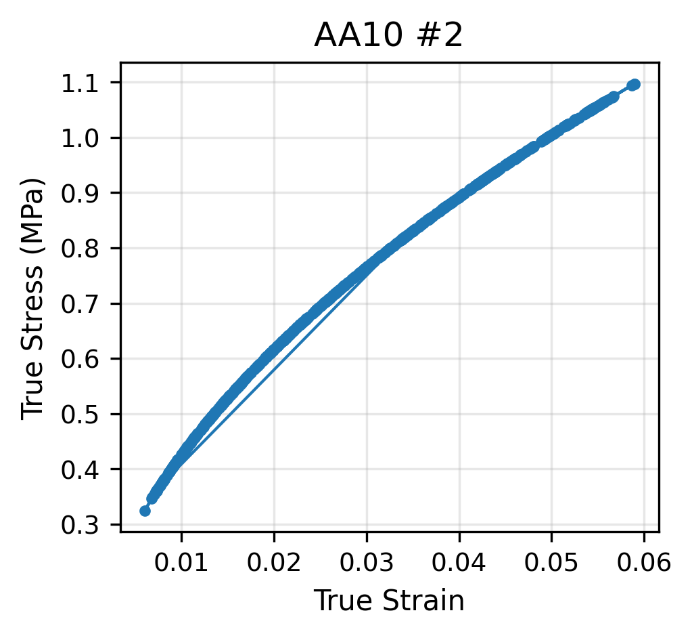

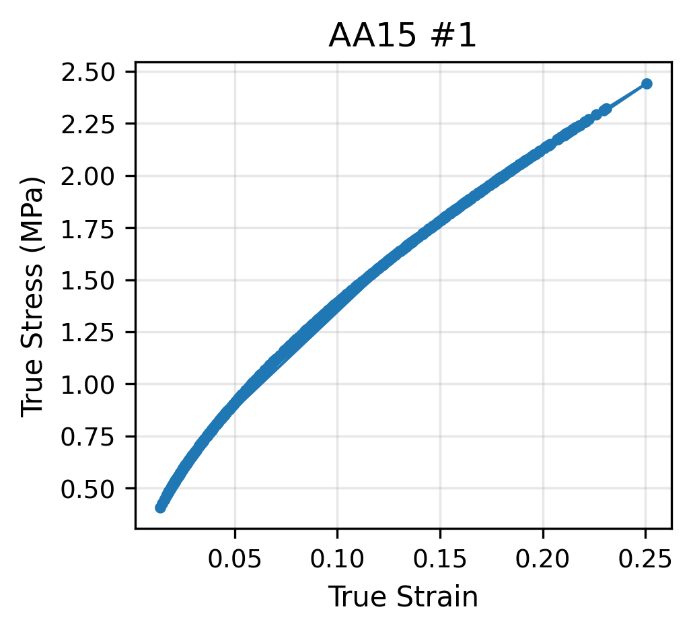


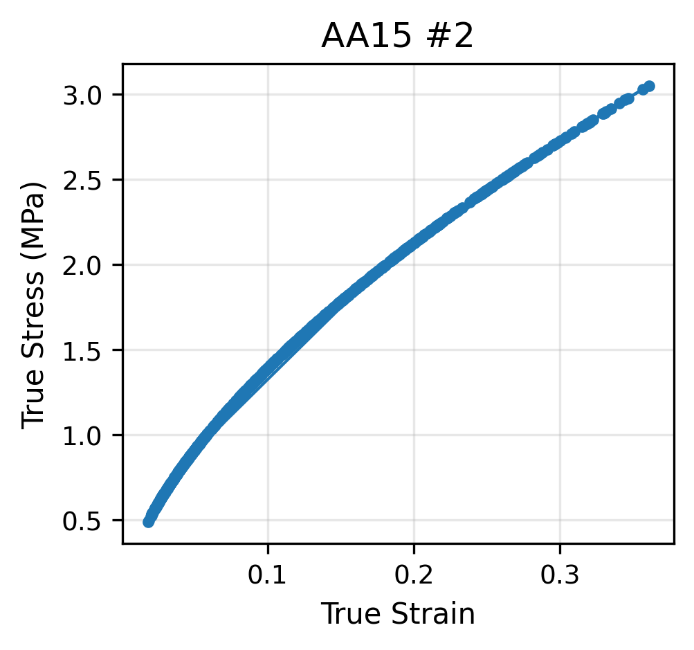

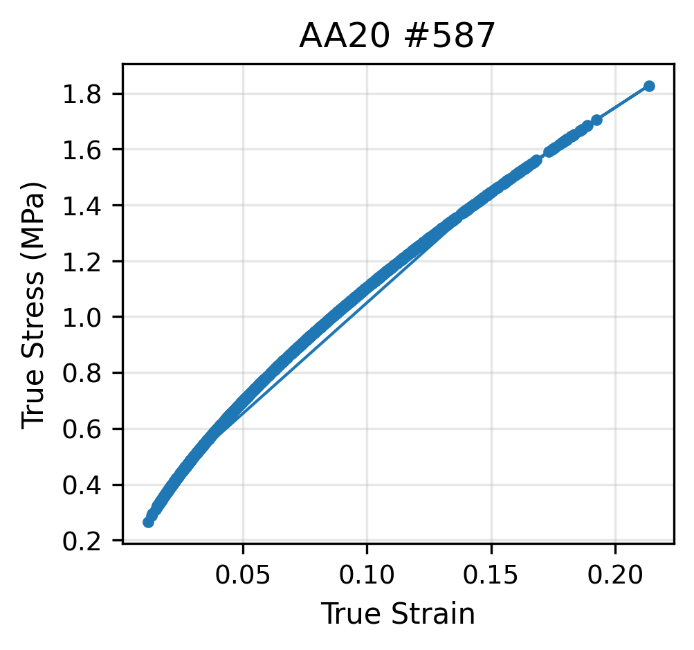


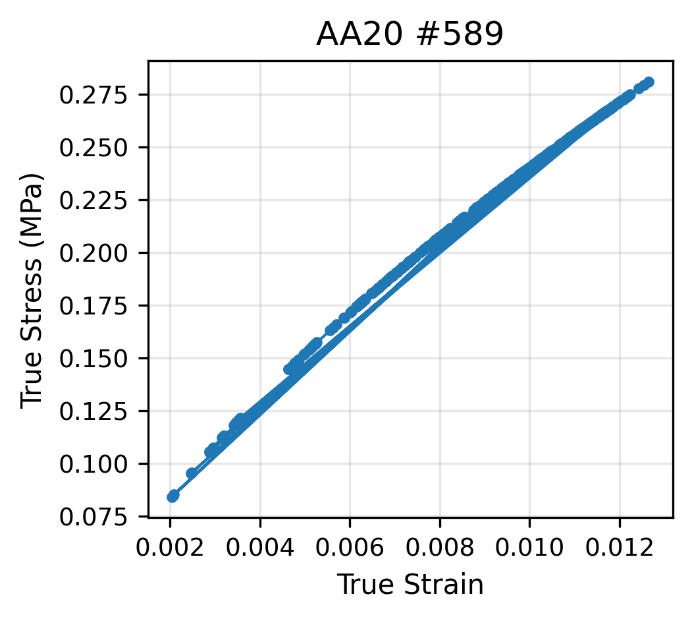


## Fatigue testing using biaxial tensile test setup

For the preliminary cyclic bulge testing, one experiment was performed for selected film types based on their expected ability to stretch and shrink. Pressure was oscillated four times between 0 and 50% of the pressure that had been determined to cause film failure during prior bulge tests. The first cycle took 210 seconds while every subsequent cycle took 180 seconds. In the fifth and final cycle, pressure was set at the maximum to cause film rupture. The air pressure was changed at rate of 0.04 bar/s.
Results with the AA-IPN films show that despite the application of the same pressure limit during the first four cycles, the highest strain observed increases slightly each cycle. This could be related to the Mullin’s effect observed in elastomers, but requires further investigation (Krpovic et al., 2021).

Table S 3 Tensile strength (MPa) comparison between single and cyclic bulge tests.

| **Film** | **Tensile strength Single test(MPa±SD)** | **Tensile strength Cyclic test (MPa)** | **Remarks** |
| --- | --- | --- | --- |
| AA5 | 10.5 (±0.9) | 12.3 |  |
| AA10 | 4.718 (±0.09) | 5.108 |  |
| AA15 | 2.61 (±0.03) | 1.48 | Broke in 2nd cycle |
| AA10-reference | 1.091 (±0.006) | 0.624 | Broke in 1st cycle |
| GC5 | 0.42 (±0.01) | 0.40 |  |


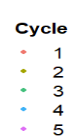

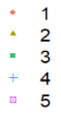

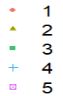

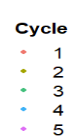

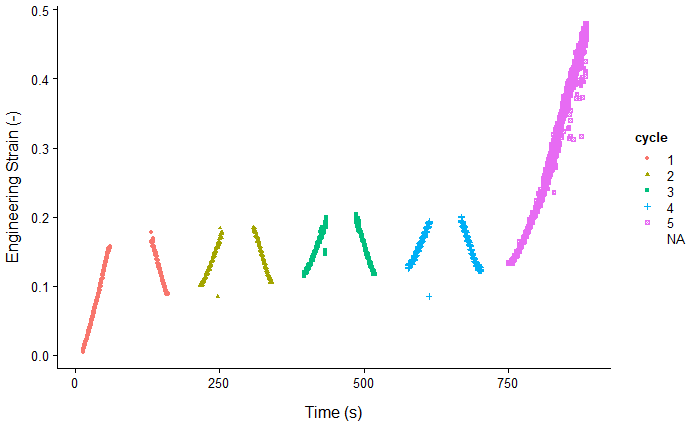

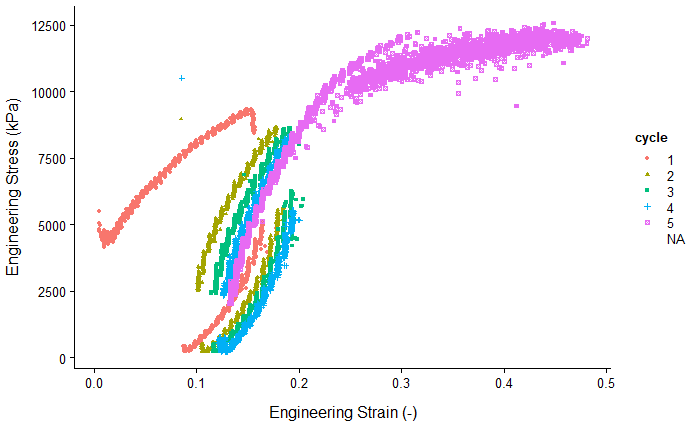


Fig. S 2 a) Engineering strain over time and b) Engineering stress-strain curve of AA5 film during a bulge test with five cycles, where maximum setpoint pressure was applied in the last cycle to cause rupture. Notably, data points during pressure calibration were removed to reduce noise.


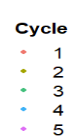

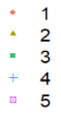

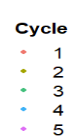

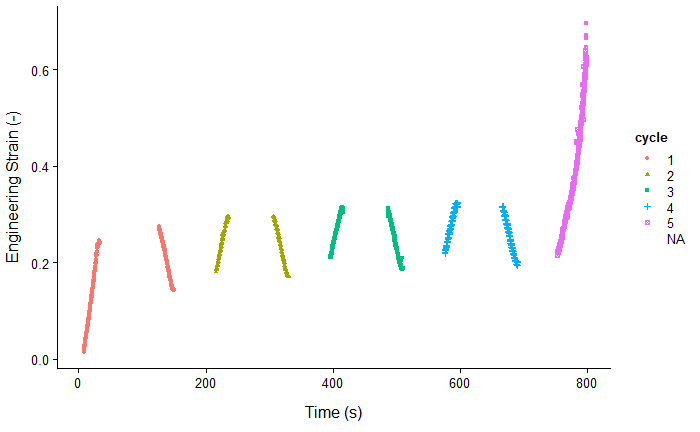

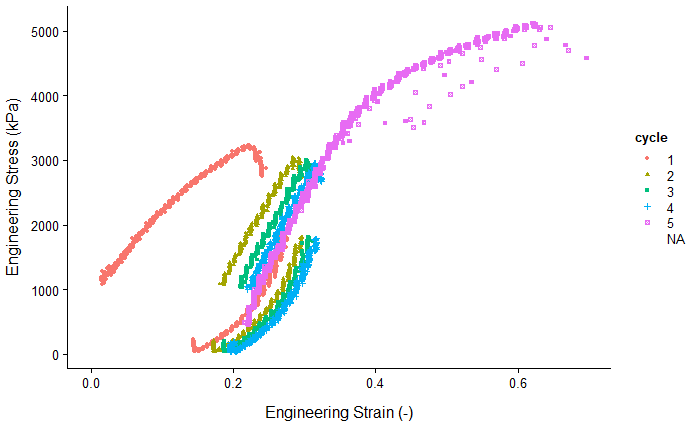
*Fig. S 3 a) Engineering strain over time and b) Engineering stress-strain curve of AA10 film during a bulge test with five cycles, where maximum setpoint pressure was applied in the last cycle to cause rupture. Notably, data points during pressure calibration were removed to reduce noise.*
