# Supplementary material for: Mechanical characterization of elastic edible films with single, crosslinked and interpenetrating biopolymer networks using combined uniaxial and biaxial analysis
Source: Curr Res Food Sci. 2026 Mar 13;12:101381. doi: 10.1016/j.crfs.2026.101381 (PMC13050118; doi:10.1016/j.crfs.2026.101381)
Supplement: Multimedia component 2 [file mmc2.pdf]

```python
import numpy as np
import matplotlib.pyplot as plt
import cv2 as cv
import pandas as pd
import time
import os
from skimage.morphology import skeletonize
from skimage.measure import EllipseModel


def ellipse_polar(angles: np.ndarray, a, b, xc=0, yc=0, theta=0):
    """Generate points for an ellipse with given parameters
    a: semi-major axis, b: semi-minor axis, theta: rotation angle"""
    x_ellipse = a * np.cos(angles)
    y_ellipse = b * np.sin(angles)

    # Apply rotation
    x = x_ellipse * np.cos(theta) - y_ellipse * np.sin(theta) + xc
    y = x_ellipse * np.sin(theta) + y_ellipse * np.cos(theta) + yc
    return x, y


def fit_ellipse(points):
    """Fit an ellipse to a set of points using scikit-image EllipseModel
    Returns: xc, yc, a, b, theta, rmse"""
    ellipse = EllipseModel()
    success = ellipse.estimate(points)

    if not success or len(points) < 5:
        return 0, 0, 0, 0, 0, float('inf')

    # Get ellipse parameters: (xc, yc, a, b, theta)
    xc, yc, a, b, theta = ellipse.params

    # Calculate RMSE
```

```python
    distances = ellipse.residuals(points)
    rmse = np.sqrt(np.mean(distances ** 2))

    return xc, yc, a, b, theta, rmse


def find_crop_dim(frame):
    """This is a general frame for deciding a good cropped area"""
    search_area = frame[530:600, 730:1245]

    """Finds the brightness value of the frame"""
    hsv = cv.cvtColor(search_area, cv.COLOR_BGR2HSV)
    brightness_channel = hsv[:, :, 2]

    """Brightness threshold to look to filter out screws, which are darker"""
    brightness_threshold = 100

    """Below the threshold becomes black with a value of 0, above is 255"""
    isTrue, bri_mask = cv.threshold(brightness_channel, brightness_threshold, 255, cv.THRESH_BINARY)

    for index, value in enumerate(bri_mask):
        """Looking for black at the left and right screws, prevents the bulge from being filtered"""
        if 0 in value[:85] or 0 in value[430:]:  # initial values were 125 and 390
            screw_index = 529 + index
            break

    """Gives the appropriate cropped area to just avoid the screws"""
    cropped_dim = (slice(200, screw_index), slice(710, 1265))
    maxy = screw_index - 200
    """Returns both the cropped dimensions and screw index which is maxy"""
    return cropped_dim, maxy
```

```python


def mask_video(frame, lower_threshold=20, upper_threshold=90):
    """Choose between the two methods below and adjust the thresholds."""

    """DIRECT CANNY EDGE DETECTION METHOD BELOW:"""

    """Conversion to grayscale"""
    gray = cv.cvtColor(frame, cv.COLOR_BGR2GRAY)

    """Applying gaussian blur"""
    blurred = cv.GaussianBlur(gray, (5, 5), 1.5)
    # was 3,3

    """Applying canny edge detection with thresholds """
    canny = cv.Canny(blurred, lower_threshold, upper_threshold)

    #     """SATURATION THRESHOLD METHOD BELOW:"""
    # #uncommented section below 4-2-25 as not a full contour was detected
    #     """Applying saturation threshold"""
    #
    #     hsv = cv.cvtColor(frame, cv.COLOR_BGR2HSV)
    #     saturation_channel = hsv[:, :, 1]
    #     saturation_threshold = 40
    #     isTrue, sat_mask = cv.threshold(saturation_channel, saturation_threshold, 255, cv.THRESH_BINARY)
    #
    #     """Erosion of features"""
    #     mask = cv.erode(sat_mask, np.ones((5, 5), "uint8"))
    #
    #     """Applying canny edge detection"""
    #     canny = cv.Canny(mask, 140, 200)

    return canny
```

```python


def find_largest_drop(lst):
    test = 0
    index = 0
    """Uses rolling sum/average to calculate"""
    for i in range(len(lst) - 9):
        sum_diff = sum(lst[i + 5:i + 9]) - sum(lst[i:i + 4])
        if sum_diff < test:
            test = sum_diff
            index = i + 4
    return index


def to_dict(*args, columns=None):
    """Writes a dictionary from the data"""
    dict = {}
    for lst, key in zip(args, columns):
        if isinstance(lst, list):
            # key = lst_name(lst)
            dict[key] = lst
    return dict


def write_to_csv(dictionary, video_filename="", lower_threshold=5, upper_threshold=60):
    """Writes the dictionary to a csv file, with the current time as filename"""

    # Create the output directory if it doesn't exist
    output_dir = "Script Output"
    if not os.path.exists(output_dir):
        os.makedirs(output_dir)

    df = pd.DataFrame.from_dict(dictionary)
    timestr = time.strftime("%Y%m%d-%H%M%S")

    # Extract just the base filename without path and extension
```

```
    if video_filename:
        base_name = os.path.basename(video_filename)
        file_name_only = os.path.splitext(base_name)[0]
    else:
        file_name_only = "unknown"

    # Include video filename and threshold values in the output filename
    # filename = f'ellipsefit_{file_name_only}_L{lower_threshold}_U{upper_threshold}_{timestr}.csv'
    filename = f'ellipsefit_{file_name_only}_L{lower_threshold}_U{upper_threshold}_PI63.csv'

    # Join the output directory with the filename
    full_path = os.path.join(output_dir, filename)

    # Save the CSV file
    df.to_csv(full_path, index=False)
    print(f"Results saved to: {full_path}")


def find_longest_list(list_of_lists):
    """This function is used to find the longest contour of the bulge"""
    longest_list = []
    max_length = 0
    for lst in list_of_lists:
        if len(lst) > max_length:
            longest_list = lst
            max_length = len(lst)
    return longest_list


def bulge_test(mov_file, pressure_file, pxcal, lower_threshold=20, upper_threshold=90, height_offset=0):
    """ In this part, the test is done, to find the x and y value of the origin of the ellipse, and the semi-axes of the ellipse"""
    accuracy = []
```

```
    semi_major = []  # Will store semi-major axis (a) for compatibility
    semi_minor = []  # Store semi-minor axis (b)
    originx = []
    originy = []
    rotation = []  # Store rotation angle
    height = []
    alpha_values = []  # Store alpha values for each frame
    va_lengths = []  # Store vector a lengths for each frame
    vb_lengths = []  # Store vector b lengths for each frame
    tme = []
    capture = cv.VideoCapture(mov_file)
    framenr = 0
    fps = capture.get(cv.CAP_PROP_FPS)
    angles = 2 * np.pi * np.linspace(0, 1, 400)

    """Selects first frame and uses it to decide the cropped dimensions"""
    isTrue, first_frame = capture.read()
    crop_dim, maxy = find_crop_dim(first_frame)

    while True:
        isTrue, frame = capture.read()
        if isTrue:
            # time stamp
            framenr += 1
            tme += [framenr / fps]

            cv.imshow('Video', frame)
            crop = frame[crop_dim]
            zeros = np.zeros(crop.shape, np.uint8)

            """Applying saturation threshold or direct canny edge detection with user-defined thresholds"""
            canny = mask_video(crop, lower_threshold, upper_threshold)

```

```
            """Dilation"""
            kernel = np.ones((5, 5), np.uint8)
            canny = cv.dilate(canny, kernel, iterations=1)

            """Skeletonising"""
            canny = skeletonize(canny).astype(np.uint8) * 255

            """Finding contours and picking longest """
            contours, hierarchy = cv.findContours(canny, cv.RETR_TREE, cv.CHAIN_APPROX_NONE)
            c = find_longest_list(contours)

            # visualization
            cv.drawContours(crop, c, -1, (0, 255, 0), 3)
            cv.imshow('Overlay', crop)  # overlay contours with image

            cv.drawContours(zeros, c, -1, (255, 255, 255), 1)
            cv.imshow('longest contour', zeros)  # contours on a dark background

            if len(c) > 15 / pxcal:
                # height
                y = c[:, 0, 1]  # all points, first color channel, second dimension
                x = c[:, 0, 0]

                h = maxy - min(y)
                h = h * pxcal + height_offset  # Add user-defined offset

                """Fit ellipse to the contour points """
                points = c[:, 0, :]  # Shape: (n_points, 2)
                xfit, yfit, afit, bfit, theta_fit,
```

```
accr = fit_ellipse(points)

                # Compute fitted ellipse curve in pixels first
                fitx_px, fity_px = ellipse_polar(angles, afit, bfit, xfit, yfit, theta_fit)

                # Convert everything to mm for plotting
                x_mm = x * pxcal
                y_mm = y * pxcal
                fitx_mm = fitx_px * pxcal
                fity_mm = fity_px * pxcal
                xc_mm = xfit * pxcal
                yc_mm = yfit * pxcal
                a_mm = afit * pxcal
                b_mm = bfit * pxcal
                rmse_mm = accr * pxcal
                h_mm = h  # already in mm

                # Peak and baseline in mm
                y_min = np.min(y)  # pixel coords
                x_peak = np.mean(x[y == y_min])
                x_peak_mm = x_peak * pxcal
                y_peak_mm = y_min * pxcal
                baseline_mm = maxy * pxcal

                # --- Find intersection of fitted ellipse with baseline ---
                # Select ellipse point closest to baseline y
                idx_base = np.argmin(np.abs(fity_mm - baseline_mm))
                x_base, y_base = fitx_mm[idx_base], fity_mm[idx_base]

                # --- Compute angle alpha between line a and line b ---
                va = np.array([x_base - xc_mm, y_base - yc_mm])
                vb = np.array([x_peak_mm - xc_mm,
```

```
y_peak_mm - yc_mm])

                # Calculate vector lengths
                va_length = np.linalg.norm(va)
                vb_length = np.linalg.norm(vb)

                dot = np.dot(va, vb)
                alpha_rad = np.arccos(dot / (va_length * vb_length))
                alpha_deg = np.degrees(alpha_rad)

                # Visualise fit for chosen frames
                if framenr in (250, 750, 1000):
                    # Compute fitted ellipse curve in pixels first
                    fitx_px, fity_px = ellipse_polar(angles, afit, bfit, xfit, yfit, theta_fit)

                    # Convert everything to mm for plotting
                    x_mm = x * pxcal
                    y_mm = y * pxcal
                    fitx_mm = fitx_px * pxcal
                    fity_mm = fity_px * pxcal
                    xc_mm = xfit * pxcal
                    yc_mm = yfit * pxcal
                    a_mm = afit * pxcal
                    b_mm = bfit * pxcal
                    rmse_mm = accr * pxcal
                    h_mm = h  # already in mm

                    # Peak and baseline in mm
                    y_min = np.min(y)  # pixel coords
                    x_peak = np.mean(x[y == y_min])
                    x_peak_mm = x_peak * pxcal
                    y_peak_mm = y_min * pxcal
                    baseline_mm = maxy * pxcal

                    # Plot contour and fitted ellipse
```

```
                    plt.plot(x_mm, y_mm, label='Contour')
                    plt.plot(fitx_mm, fity_mm, label='Ellipse Fit')

                    # Center point (x, y) as a red dot
                    plt.scatter(xc_mm, yc_mm, color="red", s=30, zorder=5, label="Center (x, y)")

                    # Draw semi-major axis
                    dx_a = a_mm * np.cos(theta_fit)
                    dy_a = a_mm * np.sin(theta_fit)
                    plt.plot([xc_mm - dx_a, xc_mm + dx_a],
                             [yc_mm - dy_a, yc_mm + dy_a],
                             linestyle="--", label="Semi-major (a)")

                    # Draw semi-minor axis
                    dx_b = b_mm * -np.sin(theta_fit)
                    dy_b = b_mm * np.cos(theta_fit)
                    plt.plot([xc_mm - dx_b, xc_mm + dx_b],
                             [yc_mm - dy_b, yc_mm + dy_b],
                             linestyle="--", label="Semi-minor (b)")

                    # Draw baseline line (clipped)
                    plt.plot(
                        [np.min(x_mm), np.max(x_mm)],
                        [baseline_mm, baseline_mm],
                        color="gray", linestyle=":", lw=1.5, label="Baseline"
                    )

                    # Draw height as vertical arrow from baseline to peak
```

```
                    plt.annotate(
                        "",
                        xy=(x_peak_mm, y_peak_mm),  # top (bulge peak)
                        xytext=(x_peak_mm, baseline_mm),  # bottom (flat baseline)
                        arrowprops=dict(arrowstyle="<->", color="purple", lw=2)
                    )

                    # Mark bulge peak with a purple dot
                    plt.scatter(x_peak_mm, y_peak_mm, color="purple", s=40, zorder=6, label="Bulge peak")

                    # Label height next to the arrow
                    plt.text(x_peak_mm + 2, (y_peak_mm + baseline_mm) / 2,
                             f"h = {h_mm:.2f} mm",
                             color="purple", fontsize=9, va="center")

                    # --- Find intersection of fitted ellipse with baseline ---
                    idx_base = np.argmin(np.abs(fity_mm - baseline_mm))
                    x_base, y_base = fitx_mm[idx_base], fity_mm[idx_base]

                    # --- Compute angle alpha between line a and line b ---
                    va = np.array([x_base - xc_mm, y_base - yc_mm])
                    vb = np.array([x_peak_mm - xc_mm, y_peak_mm - yc_mm])

                    dot = np.dot(va, vb)
                    alpha_rad = np.arccos(dot / (np.linalg.norm(va) * np.linalg.norm(vb)))
                    alpha_deg = np.degrees(alpha_rad
```

```
)

                    # --- Plot line a (center → baseline intersection) ---
                    plt.plot([xc_mm, x_base], [yc_mm, y_base],
                             linestyle=":", color="blue", linewidth=2,
                             label=f"Line a ({va_length:.2f} mm)")

                    # --- Plot line b (center → bulge peak) ---
                    plt.plot([xc_mm, x_peak_mm], [yc_mm, y_peak_mm],
                             linestyle=":", color="green", linewidth=2,
                             label=f"Line b ({vb_length:.2f} mm)")

                    # --- Annotate angle a at ellipse center ---
                    arc_radius = 5  # mm

                    # Calculate the starting angle for line a
                    start_angle = np.arctan2(y_base - yc_mm, x_base - xc_mm)

                    # Create arc from line a to line b (counterclockwise)
                    arc_angles = np.linspace(start_angle, start_angle + alpha_rad, 100)
                    arc_x = xc_mm + arc_radius * np.cos(arc_angles)
                    arc_y = yc_mm + arc_radius * np.sin(arc_angles)
                    plt.plot(arc_x, arc_y, color="orange", linewidth=2)

                    # Place text at the middle of
```

```
the arc
                    mid_angle = start_angle + alpha_rad / 2
                    text_x = xc_mm + arc_radius * 1.3 * np.cos(mid_angle)
                    text_y = yc_mm + arc_radius * 1.3 * np.sin(mid_angle)
                    plt.text(text_x, text_y,
                             f"α = {alpha_deg:.2f}°",
                             color="orange", fontsize=9, ha="center", va="center")

                    # --- Add ellipse fit parameters as text box (converted to mm) ---
                    param_text = (
                        f"a = {a_mm:.2f} mm\n"
                        f"b = {b_mm:.2f} mm\n"
                        f"xc = {xc_mm:.2f} mm\n"
                        f"yc = {yc_mm:.2f} mm\n"
                        f"θ = {theta_fit:.3f} rad\n"
                        f"h = {h_mm:.2f} mm\n"
                        f"α = {alpha_deg:.2f}°\n"
                        f"RMSE = {accr:.3f} mm"
                    )
                    plt.gca().text(
                        0.02, 0.98, param_text,
                        transform=plt.gca().transAxes,
                        fontsize=10,
                        verticalalignment='top',
                        bbox=dict(boxstyle="round,pad=0.3", facecolor="white", alpha=0.7)
                    )

                    # Styling
                    plt.xlabel("x (mm)")
                    plt.ylabel("y (mm)")
                    plt.legend(loc="upper right") # Moved legend to top-right
                    plt.gca().axis('equal')
```

```
                    plt.gca().invert_yaxis()
                    plt.title(f'Frame {framenr}')
                    plt.show()

                # Convert to physical units (already done above for calculations)
                afit_mm = afit * pxcal  # semi-major axis in mm
                bfit_mm = bfit * pxcal  # semi-minor axis in mm
                xfit_mm = xfit * pxcal  # x center in mm
                yfit_mm = yfit * pxcal  # y center in mm
                accr_mm = accr * pxcal  # RMSE in mm

            else:
                # When no contour is found, choose 0. This prevents errors
                h, xfit_mm, yfit_mm, afit_mm, bfit_mm, theta_fit, accr_mm, alpha_rad, va_length, vb_length = 0, 0, 0, 0, 0, 0, 0, 0, 0, 0

            height += [h]
            semi_major += [afit_mm]  # now in mm
            semi_minor += [bfit_mm]  # now in mm
            originx += [xfit_mm]  # now in mm
            originy += [yfit_mm]  # now in mm
            rotation += [theta_fit]
            accuracy += [accr_mm]  # RMSE in mm
            alpha_values += [alpha_rad]  # Store alpha for each frame
            va_lengths += [va_length]  # Store va length for each frame
            vb_lengths += [vb_length]  # Store vb length for each frame

            # Exit prematurely (don't use this it will break time shift correction)
            if cv.waitKey(1) & 0xFF == ord('d'):  # to be able to end the video, press d
```

```python
                print('stop condition 2')
                break

        else:
            print('stop condition 1')
            break

    capture.release()
    cv.destroyAllWindows()

    # Build a dataframe with relevant parameters
    df = pd.read_csv(pressure_file, header=None, names=['t', 'Pout', 't2', 'SetPout', 't3', 'Pin', 't4', 'SetPin'],
                     sep=';')

    """/50 is default to convert to 2 bar, /(100/6) for 6 bar"""
    df['Pin'] /= 50  # % -> bar
    df['Pout'] /= 50
    # df['Pin'] /= (100/6)
    # df['Pout'] /= (100/6)

    print(df)

    # correct for time shift
    pin = df['Pin'].to_list()
    tpop1 = df.loc[find_largest_drop(pin), 't']  # t at maximum pressure

    tpop2 = tme[find_largest_drop(height)]  # t at maximum height
    shift = tpop2 - tpop1
    df['t'] += shift  # shift pressure time to have the 'pop' at the same moment

    # interpolate pressure data
    xp = df['t'].to_list()
    paverage = df['Pin'].add(df['Pout'])
    paverage /= 2

```

```
    fp = paverage.to_list()
    pressure = np.interp(tme, xp, fp)
    pressure = pressure.tolist()

    # Calculate A3D using ellipse approximation
    # For an ellipse bulge, we approximate the 3D surface area
    # Using the formula for surface area of revolution of an ellipse
    a3d = []
    for i in range(len(semi_major)):
        if semi_major[i] > 0 and semi_minor[i] > 0 and height[i] > 0:
            # Approximation: 2 * pi * average_radius * height
            avg_radius = (semi_major[i] + semi_minor[i]) / 2
            area = 2 * np.pi * avg_radius * height[i]
            a3d.append(area)
        else:
            a3d.append(0)

    print('A3D =', a3d)

    # Fixed column order as requested with va and vb lengths added
    columns = ['t (s)', 'P (bar)', 'h (mm)', 'a (mm)', 'b (mm)', 'A3D (mm2)', 'theta (rad)', 'xc', 'yc', 'alpha (rad)',
               'va (mm)', 'vb (mm)', 'RMSE']

    # Fixed data order to match columns with va and vb lengths added
    d = to_dict(tme, pressure, height, semi_major, semi_minor, a3d, rotation, originx, originy, alpha_values,
                va_lengths, vb_lengths, accuracy, columns=columns)
    write_to_csv(d, mov_file, lower_threshold, upper_threshold)
```

```python

    for k, v in d.items():
        print(k, v)


if __name__ == "__main__":
    mov_file = 'Input Videos\\G23 00553.MTS'
    pressure_file = 'Pressure Data\\G23 vid00553.txt'
    pxcal = 0.1118  # mm/pixel
    height_offset = 0  # mm - offset added to height measurements
    lower_threshold = 20
    upper_threshold = 90
    bulge_test(mov_file, pressure_file, pxcal, height_offset=height_offset)
```
